# Evolving concerns: A longitudinal content analysis of free-text reports from the UK COVID-19 Public Experiences (COPE) study cohort

**DOI:** 10.1371/journal.pone.0348308

**Published:** 2026-11-10

**Authors:** Rhiannon Phillips, Fiona Wood, Anna Torrens-Burton, Clare Glennan, Paul Sellars, Sherina Lowe, Aleysha Caffoor, Britt Hallingberg, David Gillespie, Victoria Shepherd, Wouter Poortinga, Karin Wahl-Jorgensen, Denitza Williams

**Affiliations:** 1 Cardiff School of Sport and Health Science, Cardiff Metropolitan University, Cardiff, United Kingdom; 2 Division of Population Medicine, School of Medicine, Cardiff University, Cardiff, United Kingdom; 3 Wales Centre for Primary and Emergency Care Research, Cardiff University, Cardiff, United Kingdom; 4 Centre for Trials Research, Cardiff University, Cardiff, United Kingdom; 5 School of Psychology Cardiff University, Cardiff, United Kingdom; 6 Welsh School of Architecture, Cardiff University, Cardiff, United Kingdom; 7 Cardiff School of Journalism, Media and Culture, Cardiff University, Cardiff, United Kingdom; Dental Hypothesis, IRAN, ISLAMIC REPUBLIC OF

## Abstract

**Objectives:**

Concerns are psychological appraisals of risk that can influence how people respond to threats, including those posed by infectious diseases. The aim of this study was to characterise the evolution of concerns reported by a community cohort during the first two years of the COVID-19 pandemic.

**Design:**

Content analysis of qualitative descriptions of concerns in a prospective longitudinal online survey (COVID-19 UK Public Experiences (COPE) Study).

**Method:**

At baseline (March/April 2020), when the UK entered its first national lockdown, 11,113 adults completed the COPE survey. Follow-up surveys were conducted at 3, 12, 18 and 24 months. Participants were recruited via the HealthWise Wales research registry and social media. Baseline surveys collected demographic and health data. All waves included an open-ended question about COVID-19 concerns. Content analysis was used to identify the frequency of concerns coded to each category and number of individuals reporting concern(s) within each category at each survey wave, as participants could report multiple concerns.

**Results:**

Across five survey waves, a total of 22,971 open-text responses were received. From these, 41,569 concerns were identified and coded into six main categories of concerns: personal harm (n = 16,353 concerns coded to this category), harm to others (n = 11,464 concerns), social/economic impact (n = 6,443 concerns), preventing transmission (n = 4,838 concerns), government/media (n = 1,048 concerns), and general concerns (n = 1,423 concerns). The proportion of respondents reporting any concern declined from 75.3% at baseline to 65.8% at 24-months. Over time, the proportion of respondents reporting concerns about personal harm increased (baseline 41.8% vs. 24-months 52.7%), whereas the proportion reporting concerns about harm to others decreased (baseline 48.5% vs. 24-months 28.6%).

**Conclusions:**

These findings highlight the dynamic and multi-dimensional nature of concerns reported in this cohort during the COVID-19 pandemic. Monitoring specific concerns longitudinally during pandemics could provide valuable insights to inform communication strategies and development of public health interventions.

## Introduction

During pandemics, risk perceptions are an important determinant of how people respond to infectious disease threats and are associated with engagement in preventive behaviours such as mask-wearing, hand hygiene, social distancing, and vaccination uptake [1–14]. Risk appraisal during a pandemic can range from feelings of mild concern to intense fear [15]. Both internal and external factors such as easing and tightening of lockdown restrictions and introduction of widespread vaccination influence risk perception [5,7,16]. Understanding how people’s concerns changed over time during the COVID-19 pandemic is vital for future pandemic planning to enable equitable and adaptive public health strategies to be developed and implemented [6].

‘Concerns’ are psychological appraisals of risk that form part of the affective aspect of risk perception, which incorporates emotional responses to a threat like worry and fear [17]. Affective risk perception works alongside, but independently of, more reflective and analytical aspects of risk perception in determining behaviour [17]. Quantitative longitudinal studies have indicated that overall perception of risk decreased over the time during the COVID-19 pandemic, corresponding with decreased engagement in protective action, but that affective risk perception was strongly associated with protective behaviour throughout [6,18]. A bi-directional relationship was observed between high perceived risk of COVID-19 and poorer mental health and well-being over the course of the pandemic [19]. Qualitative studies during the COVID-19 pandemic demonstrated that people's concerns were closely linked to their experiences, emotional well-being, perceptions of risk, and reported decision-making regarding protective measures, and could differ according to individual, demographic, contextual, cultural and geographic factors [20–24].

Monitoring evolving concerns can enable those planning pandemic-related communication to balance the need to convey seriousness with the need to prevent excessive fear or fatalism, which can erode resilience and social cohesion [25,26]. Longitudinal understanding of concerns can reveal emerging issues, such as worries about economic consequences or long-term health and well-being effects, that require policy adjustments beyond infection control alone [27–29]. Large-scale studies of risk perceptions during the COVID-19 pandemic typically relied on broad quantitative measures of susceptibility of self/others to contracting infection, severity, risk of infecting others, and infection-related anxiety or fear [5–7,15,21,30–33]. However, these assessments do not fully illuminate the specific concerns that underlie the affective dimension of risk perception. Conversely, qualitative studies focused on providing in-depth insights into the views and experiences of specific populations and contexts during the pandemic, rather than providing broader insight into shifts in the types of concerns reported by large community cohorts in real time. As such, it is unclear how specific concerns about COVID-19 evolved over the course of the pandemic, and how people balanced concerns about harm from infection with concerns about the wider psychological, social and economic impact of the pandemic.

While previous longitudinal studies have examined changes in perceived risk and protective behaviours using quantitative measures, considerably less attention has been given to understanding the specific concerns underpinning these perceptions. Identifying the types of concerns people express, and how these evolve over time, can provide insight into changing priorities, perceived threats, and competing needs during a prolonged public health emergency. Affective risk perceptions have been associated with infection-prevention behaviours during the COVID-19 pandemic, but concerns themselves represent a distinct psychological construct. Understanding the nature of people's concerns is important in its own right, regardless of whether subsequent behaviours are measured directly.

The aim of this study was to analyse free-text qualitative responses from a large longitudinal UK community cohort to characterise the nature, relative frequency, and evolution of concerns expressed by participants during the first two years of the COVID-19 pandemic. The study was designed to provide a detailed understanding of specific concerns over time rather than to examine behavioural outcomes directly, complementing existing quantitative evidence.

## Method

### The COVID-19 Public Experiences (COPE) Study

Data included in this analysis were collected as part of the COVID-19 Public Experiences (COPE) project [5,34,35]. COPE was a longitudinal mixed-methods study designed to examine public perceptions, experiences, and protective behaviour during the COVID-19 pandemic in the UK. The study comprised five online surveys conducted over a 24-month period between March 2020 and April 2022. Analysis of the quantitative survey data indicated motivational variables (fear of COVID-19, perceived susceptibility and perceived control) fluctuated over time as the disease and socio-political environment changed, decreasing overall by 24 months [6]. Higher levels of fear, older age, lower perceived personal control over infection transmission, more trust in government and less trust in social media were associated with more infection–prevention behaviour [6]. The current analysis extends previous work by providing a more in-depth understanding of specific concerns underpinning the observed quantitative changes in motivation over time. The COPE study received ethical approval from Cardiff Metropolitan University Applied Psychology ethics panel on 13.3.20 (Project reference Sta-2707).

### Theoretical approach

A social phenomenological approach was adopted in this study, using a data-driven process of understanding how people make sense of and interpret phenomena in their everyday world [36]. We drew on established behaviour change theory to guide analysis and facilitate interpretation of data. Concerns, as part of affective risk perception is part of the ‘automatic’ component of motivation within the Capability, Opportunity and Motivation (COM-B) model of behaviour, which includes instinctive, drive-related and affective processes such as desires, fears and habits. The Plans, Responses, Impulses, Motives, Evaluations (PRIME) Theory of Motivation [16], elaborating on the motivational component of the COM-B, proposes that behaviour at any one moment arises from the strongest of potentially competing impulses and inhibitions [16]. PRIME Theory postulates that affective motivation has a direct effect on behaviour but also influences behaviour via its impact on reflective motivation, which involves conscious decision-making, by creating a need or desire to plan and enact a particular behaviour [12]. PRIME theory was relevant to developing theory-informed approaches to increase adherence to preventative measures during the COVID-19 pandemic [12]. In the present study, we sought to use PRIME Theory and COM-B as a guide in developing insight into how different concerns, needs and impulses were balanced at different points in time.

### Study population and recruitment

A total of 11,113 adults living in the UK participated in the COPE study at the time of enrolment, which took place during the first national lockdown (13/03/2020 to 13/04/2020) [34,35]. An early lineage of the wildtype SARSCoV2 carrying the D614G mutation was the dominant variant circulating in the UK at this time. Participants were recruited through social media advertisements (Facebook®, Twitter®, and Instagram®) and via the HealthWise Wales (HWW) research registry [37]. The sample size was determined using a pragmatic approach, seeking to recruit sufficient numbers of people to enable us to understand a wide range of experiences in different geographical regions (urban/semi-rural/rural and varying levels of socio-economic deprivation) and demographic groups, allowing for attrition over time, while considering resources available and the need for a flexible and responsive approach to data collection in an unpredictable and rapidly changing context. At baseline, the majority of the 11,113 participants were female (69.2%), aged 51 years or older (68.3%), white British (95.8%), had a pre-existing medical condition (50.5%), and had received college (post-18) education (67.1%) [35]. Further detail of demographic characteristics is provided in S1 Table.

Follow-up surveys took place at the following time points:

- 3-months, 20/06/2020 to 20/07/2020, n = 7,048, wildtype D614G variant dominant in UK, gradual easing of UK lockdown following the first wave of the pandemic
- 12-months, 12/03/2021 to 13/04/2021, n = 5,535, Alpha variant dominant in UK, gradual easing of lockdown restrictions in UK after a second full UK-wide lockdown that had come into force in January 2021, a UK-wide COVID-19 mass vaccination program had commenced
- 18-months, 28/09/2021 to 03/11/2021, n = 4,242, Delta variant dominant in UK, most UK COVID-19 restrictions lifted and most UK adults had received at least one vaccination against COVID-19
- 24-months, 22/03/2022 to 28/04/2022, n = 3,827, Omicron variant dominant in UK, remaining COVID-19 restrictions being removed, UK government communications moved towards a ‘new normal’ where COVID-19 had become endemic

### Materials and measures

The qualitative data analysed in this paper were participants’ responses to an open-ended question included in each of the COPE surveys. Free-text responses were collected at all five survey waves using the prompt:

*“If you are worried about coronavirus (COVID-19), please tell us what your main concerns are.”*

The prompt was structured in this way so as not to assume that survey respondents were worried or had concerns about COVID-19, enabling people to report any salient concerns that they may have in their own words. These qualitative responses were analysed to explore how risk perceptions and related concerns evolved over time. The baseline survey also collected demographic and health-related data, including age, gender, highest educational qualification, ethnic group, presence of children under 18 years in the household, pre-existing medical conditions, and seasonal influenza vaccination within the preceding 12 months.

### Analysis

Content analysis was performed on open-text responses relating to what people’s main concerns were (if they had any) at each time point [30]. This approach enabled efficient analysis of large quantities of text into categories with similar meanings, allowed interpretation of meaning from the content and context of free- text using a naturalistic paradigm, and provided a quantitative indication of the prevalence of categories of concerns [30]. The purpose of this analysis of qualitative data is to provide rich, contextualised insights into the nature and relative prominence of concerns reported by this cohort. The study is not designed to produce quantitative estimates of prevalence or findings that are generalisable at the population level.

An inductive analytic process was applied to the baseline data that involved familiarisation, generation of initial codes, combining codes into categories, reviewing categories, and determining significance of categories to generate a coding framework for the content analysis. The coding framework developed using the baseline data was applied deductively to the follow-up survey data to allow consistency in coding across time points. This was supplemented with further inductive analysis of concerns that did not fit into existing categories, producing new categories as required as the pandemic progressed.

Text responses were coded by a team of 11 researchers. To enable effective team-based coding, a clear and supportive management structure was in place, the team met regularly to discuss analysis and interpretation, detailed guidance and reference materials (codebook, written guidance and training materials) were provided, and coders were supported in building up skills gradually [38]. Comments were coded in Microsoft Excel to accommodate the volume of data and coding by multiple researchers. Comments could be coded into multiple categories if required.

As interpretation is influenced by researchers’ backgrounds and disciplinary perspectives, we considered transparency regarding the composition of the coding team important as part of a reflexive qualitative approach. Coders were an inter-disciplinary team with expertise in psychology, medical sociology, nursing, palliative care, biosciences, public health and health services research, were all females of working age, with a variety of social backgrounds and family situations. The coders were resident in Wales at the time the study was being conducted and were experiencing many similar challenges in living and working through the pandemic as the study participants, along with a variety of issues relevant to their own personal situations. The team-based approach to analysis was vital in allowing discussion and reflection on the potential impact of researchers’ own views and experiences on the analytic process.

A random sample of 10% of the baseline data were verified by a second member of the research team (RP) to check the consistency and accuracy of coding across the team. The same coding framework was applied by all the coders, with ongoing coding queries and borderline cases discussed and resolved at regular team meetings throughout. No formal agreement statistics were calculated. Our quality assurance approach was instead based on team-based qualitative coding procedures, including detailed coding guidance, coder training, regular meetings, second-review verification of a random 10% sample, and independent review of the general concerns category (RP/ATB) to assess whether they could be re-coded within one of the existing codes and to identify the need for new codes. Disagreements between coders were resolved through discussion and consultation with a third coder if required. Comments written in Welsh were coded directly from the text by a bilingual Welsh/English member of the coding team (RP). On completion of coding and quality assurance, the frequency of codes assigned to each main category and sub-category at each time point was counted [30]. These frequencies represent the number of coded concerns and not the number of participants, as multiple codes could be assigned to a response form a participant where multiple concerns were reported. Further, the analysis approach highlights differences in the prominence of categories of concerns reported at each survey wave as opposed to tracking changes at an individual level.

The primary aim of this analysis was not to test hypotheses regarding predictors of concern categories or to statistically model longitudinal predictors of concern categories, but rather to provide an in-depth understanding of the nature and evolution of concerns expressed in participants’ own words over the first two years of the pandemic. Consistent with the analysis approach, coding frequencies were used descriptively to illustrate the relative frequency of qualitatively-derived categories within the dataset rather than as outcome variables for inferential statistical modelling. Exploratory descriptive analyses were undertaken to examine whether the prominence of the main concern categories differed according to selected baseline participant characteristics (S4-S6 Tables). These analyses were intended to provide contextual insight rather than formal subgroup comparisons of categories of concerns or formal subgroup differences.

## Results

Across the five survey waves, a total of 22,971 open-text responses were received, describing participants main concerns about COVID-19. Concerns were provided by 8,372 of 11,113 (75.3%) people at baseline, 5,330 of 7,048 (75.6%) at three-months, 3,823 of 5,535 (69.1%) at 12-months, 2,928 of 4,242 (69.0%) at 18-months, and 2,518 of 3,827 (65.8%) at 24-months. This overall reduction in the proportion of people reporting concerns was consistent with the overall decrease in fear of COVID-19 observed using quantitative data from the COPE cohort [6].

Participants could report multiple concerns within their responses, and as such 41,569 concerns were identified over the course of the study. The six main categories of concerns identified through content analysis were: 1. Personal harm (n = 16,353 concerns coded to this category); 2. Harm to others (n = 11,464 concerns; 3. Social and economic impacts (n = 6,443 concerns); 4. Preventing the spread of COVID-19 (n = 4,838 concerns); 5. Government and media (n = 1,048 concerns), and 6. General concerns (n = 1,423 concerns).

Fig 1 illustrates the change in percentage of participants reporting one or more concerns in each main category at each survey. Percentages are based on respondents and therefore differ from the category frequencies reported above. In interpreting these findings, it should be noted that frequency of reporting of specific concerns is not a direct measure of psychological importance or emotional intensity. Rather, they indicate how commonly specific concerns were mentioned within participants’ responses across each survey wave. Less frequently occurring concerns could nevertheless have profound significance for the individuals reporting them.

**Fig 1:**
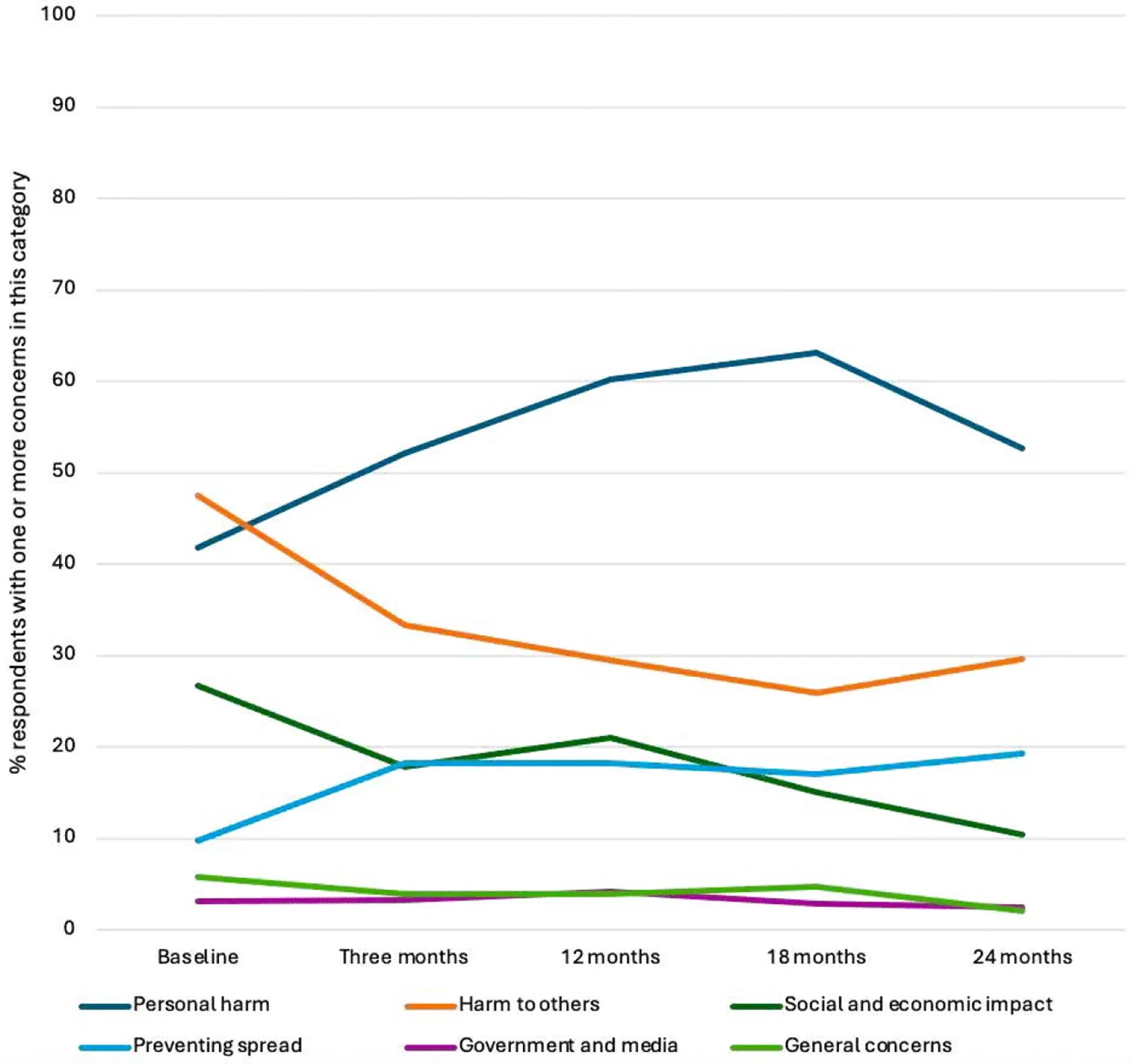
Percentage of respondents reporting main categories of concerns at each survey time point.

Each main category is further divided into sub-categories, which capture the breadth and nuance of participants’ concerns across five survey time points, illustrating how priorities and fears shifted over the two-year study period. A total of 30 sub-categories were identified (Table 1). Individuals who provided a description of their concerns in each survey reported between one and 10 sub-categories of concern, with the median being two sub-categories of concerns reported by individuals at baseline, 12-months and 18-months, and a median of one sub-category of concerns at 3-months and 24-months. A table of exemplar quotes in main and sub-categories at each survey time point is provided in S2 Table.

**Table 1:** Number of participants and % reporting concern(s) in each sub-categories at each survey time point.

Main Category	Sub-category	Description	Baseline N = 11,113		Three months N = 7,048		12-months N = 5,535		18-months N = 4,242		24-months N = 3,827	
			N	%	N	%	N	%	N	%	N	%
**Personal harm** *Worry about individuals’ own health and vulnerability to infection*	Serious physical harm/death	Becoming seriously ill, being hospitalised and/or dying due to COVID-19	1575	14.2	1010	14.3	881	15.9	584	13.8	361	9.4
	Becoming infected	Concerns about contracting COVID-19 (no specific mention of vulnerability or serious harm)	1206	10.9	777	11.0	668	12.1	527	12.4	334	8.7
	Clinical vulnerability	Perceived vulnerability due to underlying condition, age, or other relevant factor	937	8.4	1084	15.4	587	10.6	505	11.9	564	14.7
	How COVID will affect me/my family	Uncertainty about potential effects and unpredictability of harm/impact from infection to individuals.	566	5.1	443	6.3	558	10.1	481	11.3	174	4.5
	Impact on caring responsibilities	How people or animals an individual cares for would be affected if they were unable to provide care due to COVID-19	307	2.8	167	2.4	123	2.2	494	11.6	59	1.5
	Long-term harm	Concerns about long-term harm from COVID-19 or ‘Long-Covid’	50	0.4	200	2.8	523	9.4	494	11.6	524	13.7
**Harm to others** *Concerns for family members, loved ones, and wider society*	Family or loved ones becoming infected	Concerns about family or loved ones contracting COVID-19 (no specific mention of vulnerability or serious harm)	1601	14.4	544	7.7	564	10.2	311	7.3	325	8.5
	Clinical vulnerability	Vulnerability of family or loved ones to harm from COVID-19 infection due to underlying condition, age, or other relevant factor	1361	12.2	723	10.3	315	5.7	302	7.1	335	8.8
	Serious physical harm	Family or loved ones becoming seriously ill, being hospitalised and/or dying due to COVID-19	1290	11.6	469	6.7	355	6.4	200	4.7	89	2.3
	Vulnerable people in society	Concerns about harm from COVID-19 to vulnerable people more generally (i.e., beyond family/loved ones)	382	3.4	84	1.2	79	1.4	72	1.7	109	2.8
	Transmitting the virus to others	Concerns about transmitting COVID-19 to other people, including those who may be more vulnerable to harm, and possibility of unknowingly passing on infection.	649	5.8	533	7.6	319	5.8	215	5.1	238	6.2
**Social and economic impacts** *Perceived disruption to daily life, employment, education, and mental well-being*	Society and economy	Wider impact on economy and infrastructure, e.g., goods, travel, global impact, shift in socio-cultural norms.	853	7.7	359	5.1	279	5.0	140	3.3	67	1.8
	Daily activities	Impact of the pandemic on activities such as leisure, holidays, socialising, daily activities.	722	6.5	89	1.3	140	2.5	78	1.8	89	2.3
	Healthcare services	Ability of National Health Service (NHS) to cope with COVID-19, impact of COVID-19 on ability to deliver other services, and impact of public health interventions on service delivery	647	5.8	75	1.1	64	1.2	104	2.5	68	1.8
	Work and finances	Concerns about impact of the pandemic on work and personal/family finances	573	5.2	245	3.5	129	2.3	54	1.3	57	1.5
	Social isolation	Concerns about being alone (including coping while unwell if alone) or not being able to see family and loved ones due to infection, social distancing and/or travel restrictions	376	3.4	273	3.9	244	4.4	136	3.2	67	1.8
	Mental health	Impact of the COVID-19 pandemic on emotional well-being and mental health of self and others	232	2.1	124	1.8	126	2.3	53	1.2	15	0.4
	Children and education	Impact on children and young people, including development, well-being, education and schools	105	0.9	94	1.3	80	1.4	64	1.5	36	0.9
**Preventing spread of COVID-19** *Concern about infection control measures, compliance with public health guidance, and changing restrictions*	Others not following rules	Other people not conforming to restrictions and guidelines, perceived lack of consequences/injustice for those breaking rules particularly prominent public figures	431	3.9	556	7.9	347	6.3	299	7.0	143	3.7
	Health protection interventions	Vaccines, tests and cures/treatment	256	2.3	164	2.3	246	4.4	164	3.9	189	4.9
	Lack of protection at work	Lack of PPE, policies, and procedures at work to provide protection	352	3.2	59	0.8	42	0.8	28	0.7	20	0.5
	New waves and variants	Concerns about further waves and new variants of COVID-19, including perceptions that cases are increasing in the community	28	0.3	222	3.1	150	2.7	111	2.6	238	6.2
	Asymptomatic spread	Concerns about spread via asymptomatic cases	12	0.1	96	1.4	60	1.1	44	1.0	23	0.6
	Increasing restrictions	Prospect of return to lockdown measures and/or need for return of or new restrictions to reduce spread	11	0.1	44	0.6	52	0.9	20	0.5	11	0.3
**Government and media** *Trust, communication, and perceptions of governmental and media responses*	Decreasing restrictions	Concerns about easing or removal of lockdown restrictions, risks associated with increased	1	0.0	140	2.0	109	2.0	61	1.4	114	3.0
	Government concerns	Trust in government, civil liberties, political agenda, (lack of) consistency between UK nations	348	3.1	198	2.8	209	3.8	100	2.4	87	2.3
	Media concerns	Trust in media, extent of coverage of COVID-19, reliability of information, regional relevance of coverage	12	0.1	37	0.5	28	0.5	22	0.5	7	0.2
**General concerns** *Broader feelings of uncertainty and apprehension about the future trajectory of the pandemic*	Non-specific	Non-specific concerns that cannot be categorised elsewhere, e.g., ‘this is going to be bad’	267	2.4	113	1.6	47	0.8	107	2.5	44	1.1
	Timeline	General concerns/uncertainty about what will happen in the future and whether/when things will return to ‘normal’	243	2.2	122	1.7	151	2.7	69	1.6	23	0.6
	Uncertainty	General concerns about the unpredictability or uncertainty relating to COVID-19 and its impact on individuals and society	131	1.2	43	0.6	20	0.4	28	0.7	15	0.4

NB: Participants could report multiple concerns within a single response and therefore be coded into multiple categories and sub-categories. The counts shown represent the number of participants reporting concern(s) in each category, and category totals may exceed 100%.

Exploratory descriptive analysis of the mean number of concerns coded into each main category reported across survey waves is provided as supplementary material (S4-S6 Tables). This analysis indicated that older adults (≥61 years) tended to report a greater number of concerns relating to personal harm from COVID-19 and fewer concerns relating to harm to others and wider social and economic impacts than younger adults. Women generally reported fewer concerns relating to personal harm and government/media issues, but more concerns relating to harm to others, wider social and economic impacts, and preventing the spread of COVID-19 compared with men. Participants reporting one or more pre-existing medical conditions at baseline tended to report more concerns relating to personal harm, but fewer concerns relating to harm to others, wider social and economic impacts, and general concerns than those reporting no pre-existing conditions. These patterns were broadly consistent across time points but should be interpreted descriptively given the qualitative design of the study.

### Personal harm from COVID-19

Personal harm from COVID-19 was the most common concern at all time points, with one or more concerns within this category reported by 4,641/11,113 (41.8%) respondents at baseline, 3,681/7,048 (52.2%) at 3-months, 3,340/5,535 (60.3%) at 12-months, 2,675/4,242 (63.1%) at 18-months, and 2,016/3,827 (52.7%) at 24-months. Early in the pandemic, concerns centred on severe illness, hospitalisation, breathlessness, and death were prominent.

*“I’m worried about being in pain and maybe being in hospital alone. I’m worried about dying and leaving my family.”*

(P1, female, age 41-50, pre-existing condition(s), baseline)

Despite ongoing concern about personal harm from COVID-19, fears about serious harm or death decreased over time (14.2% of respondents reporting concern(s) in this sub-category at baseline vs. 9.4% at 24 months).

Concerns about clinical vulnerability peaked at three and 24 months, when population-level government protections were being reduced/removed. Clinical vulnerability was a major driver of perceived personal risk, particularly among those with existing health conditions.

*“I've been terrified that Covid could kill me (I've been in ICU previously with my asthma)”*

(P2, female, age 31–40, pre-existing condition(s), 12-months)

Participants worried about contracting COVID-19 even without severe consequences, due to the unpleasant experience of illness and disruption to daily life.

*“Although I did not require hospital treatment, COVID-19 completely floored me. I was terribly poorly for a couple of weeks and then continued to suffer with the longest lasting 'flu' I've ever experienced.”*

(P4, female, age 51–60, no pre-existing condition, 24-months)

The perceived unpredictability of the illness heightened concern about personal and familial outcomes.

*“No two people are affected in the same way and no way of knowing how the infection would develop in myself & family.”*

(P5, female, age 71-80, pre-existing condition(s), 18 months)

Over time, worries about long-term health consequences, particularly long-covid, became more prominent as people balanced reduction in restrictions with ongoing risks of infection.

*“The number of cases going up and people starting to mix I am concerned that either I, or more vulnerable friends and family, will get Covid and be very ill or get long-covid. We can't stay cooped up forever, but it is still a dangerous disease even though we are vaccinated”*

(P6, female, age 41-50, pre-existing condition(s), 18-months)

Across all stages of the pandemic, participants worried about how illness might impair their ability to care for dependants, including children and vulnerable adults.

*“I am also concerned that my wife and I will catch it, and suffer the symptoms of it, at the same time and be unable to effectively look after our children.”*

(P7, male, age 31-40, pre-existing condition(s), baseline)

*“I am an unpaid carer for our adult autistic son. If we become unwell, there is no back up plan. If he becomes unwell, he cannot tolerate pain or hospitalisation. We have no support.”*

(P8, female, age 61–70, pre-existing condition(s), 18-months)

Concerns about harm from COVID-19 persisted for some even after vaccination, indicating that immunisation reduced but did not eliminate perceived threat.

*“I have severe asthma and bronchiectasis. Although I am double vaccinated and getting my booster on November 5* ^*th*^ *, I am still too nervous to go outside unless I really have to.”*

(P3, female, age 61–70, pre-existing condition(s), 18 months)

*“The fact that it [COVID-19] has not gone away and even though I have been fully vaccinated I know I could catch it and be fairly ill.”*

(P34, female, age 71–80, pre-existing condition(s), 24 months)

### Harm to others from COVID-19

Concerns about harm to others declined over time, with 5,283/11,113 (47.5%) of respondents reporting one or more concern in this category at baseline, 2,353/7,048 (33.4%) at 3-months, 1,632/5,535 (29.5%) at 12-months, 1,100/4,242 (25.9%) at 18-months, and 1,096/3,827 (28.6%) at 24-months. Early worries focused on family members or loved ones becoming seriously ill or dying, particularly those perceived as clinically vulnerable due to age or pre-existing conditions.

*“My main concern is not for me, I am fairly fit and healthy and had a dose of a fluey/coldy bug before Christmas, I am very afraid for my partner considering that the last bout of flu that he had, he ended up in hospital for three weeks due to pneumonia and a heart attack induced by the pneumonia.”*

(P10, female, age 41-50, no pre-existing conditions, baseline)

*“A terrible death for myself and my family members.”*

(P11, male, age 61–70, pre-existing conditions, 12 months)

Participants also worried about transmitting the virus to vulnerable relatives or partners.

*“My concern is catching it and passing it on to my partner as he is in a high-risk category. I think I would be ok but he could genuinely die.”*

(P13, female, age 41-50, no pre-existing conditions, baseline)

These concerns were intensified by perceptions that others were behaving irresponsibly and by the removal of public health protections.

*“I remain worried that I or someone I love will experience serious health problems as a result of catching the virus. I believe that with the end of free testing and the push to return to normality I have very little control now over exposure to infection.”*

(P12, male, age 41-50, no pre-existing condition, 24-months)

Beyond immediate family, concerns for vulnerable groups within communities and globally were commonly reported.

*“Worried about the weakest members of my community, worried about local business closing down, worried about permanent damage the virus might cause”*

(P14, female, age 31-40, pre-existing condition, three-months)

### Social and economic impacts

Participants reported a wide range of concerns about the broader societal consequences of the pandemic, with 2,971/11,113 (26.7%) respondents at baseline reporting a concern in this category, 1,259/7,048 (17.9%) at 3-months, 1,164/5,535 (21.0%) at 12-months, 640/4,242 (15.1%) at 18-months, and 399/3,827 (10.4%) at 24-months, indicating that these concerns were more common during periods when restrictions were more stringent. At baseline, worries focused on infrastructure and access to essential supplies, especially during periods of panic buying and potential self-isolation.

*“I worry not about the illness but about the way people are shutting themselves away and panic buying food. It will be a disaster for an already fragile economy.”*

(P15, female, age 51-60, pre-existing condition(s), baseline)

Concerns extended to the wider social, economic, and cultural impacts of COVID-19 and associated public health interventions, including effects on health workers, the economy, and vulnerable populations.

*“I'm mainly worried about the effects on society, health workers, the economy and the most vulnerable people”*

(P16, male, age 41-50, no pre-existing condition, 12 months)

*“I am very concerned at the strain the pandemic has put on health and social care workers and the way the education and social development of children and young people have been affected.”*

(P17, female, age 61-70, no pre-existing condition, 24 months)

Disruption to daily life and cancelled plans were frequently mentioned, often linked to declining mental well-being.

*“My concerns focus on the disruption to my life and the lives of the people I love. Plans for the year have been cancelled and this can have an effect on my mental health.”*

(P18, female, age 31-40, no pre-existing conditions, baseline)

Financial insecurity and employment instability were key concerns, ranging from temporary income loss to potential long-term business collapse.

*“That I will lose my business, due to an economic collapse and/or collapse of the delivery infrastructure (I run an online shop)”*

(P19, female, age 41-50, no pre-existing condition, baseline)

*“How I would financially support myself if I had to self-isolate and could not work or if I were off work due to sickness with it.”*

(P20, female, age 31–40, pre-existing condition(s), 24-months)

Concerns about the NHS focused on its capacity to manage COVID-19 cases and the implications for access to routine healthcare.

*“That the NHS won’t cope. That someone I care about will die, either because of COVID-19 or something else because hospital too full.”*

(P21, female, age 51–60, no pre-existing conditions, baseline)

*“I'm more worried about access to health care for existing chronic health conditions than I am about Covid.”*

(P22, female, age 51-60, pre-existing condition, three-months)

Social isolation was a prominent concern, especially regarding separation from loved ones and the well-being of those living alone or at high risk.

*“My biggest worries are for my family with my father and two grandparents considered high-risk for COVID19. I am worried both for their health and their mental well-being if they were to become isolated for an extended period of time.”*

(P23, male, age 18–30, no pre-existing conditions, baseline)

Geographical distance compounded these concerns for some families.

*“Our parents live 180 miles+ away in England and the Welsh Government limits us to 5 miles radius for travel. So, unless we do a ‘Dominic Cummings’, we are largely on our own, as all our other friends have children who have to social distance.”*

(P24, male, age 31-40, pre-existing condition(s), three-months)

The reference to ‘Dominic Cummings’ related to a prominent government advisor during Boris Johnson’s time as UK Prime Minister who travelled 264 miles with his family despite having symptoms of COVID-19 in May 2020, which was widely reported in the UK mainstream media [28].

Some participants reported concerns about mental health impacts, including anxiety, loneliness, and exacerbation of pre-existing conditions.

*“Concerned about pressure on NHS staff and Carers who might be suffering from PTSD. Concerned about mental well-being of friends who live alone.”*

(P25, female, age 71-80, pre-existing condition(s), three months)

Concerns about children and young people during lockdown periods focused on education, development, and well-being.

*“I am most concerned about the severe negative effects on my grandchildren's lack of education and socialising, and on the welfare of my family than I am about becoming physically unwell.”*

(P26, female, age 61-70, pre-existing condition(s) three months)

When schools reopened, worries shifted to infection risks in educational settings, particularly for clinically vulnerable families.

*“I have 4 children who have to go to school - 2 in a very large secondary and 2 clinically extremely vulnerable children whom the school and council have said they will be fined for non-attendance even though they have letters to shield till 31st March. The fallout if any of us catching Covid will be very tough, but catastrophic if I as the main person who cares for the rest of the household becomes ill.”*

(P27, female, age 41–50, pre-existing condition(s), 12 months)

### Preventing spread of COVID-19

Concerns in this category became more common as time progressed, reported by 1,091/11,113 (9.8%) respondents at baseline reporting concern(s) in this category, 1,281/7,048 (18.2%) at 3-months, 1,006/5,535 (18.2%) at 12-months, 727/4,242 (17.1%) at 18-months, and 738/3,827 (19.3%) at 24-months. For much of the pandemic, participants were worried about others not adhering to guidelines, leading to feelings of vulnerability and lack of control.

*“My continuing health effectively depends on other people doing the right things, and that isn't always going to happen.”*

(P29, female, age 41–50, pre-existing condition(s), baseline)

Non-compliance was sometimes framed in moral terms, reflecting frustration with perceived irresponsible behaviour.

*“Spread of the virus by careless, arrogant people who will not follow guidelines.”*

(P30, male, age 71–80, no pre-existing condition, 12 months)

Early concerns focused on the lack of testing, vaccines, and treatments, which heightened perceived risk for vulnerable individuals.

*“I'm worried for my parents and vulnerable friends who may not be able to pull through if they catch it as there is no vaccine or therapeutic drugs available at the moment”*

(P31, male, age 31–40, no pre-existing conditions, baseline)

*“I work with a lot of elderly and vulnerable patients and can’t get tested. I’m currently self-isolating due to high temp but will never know if I contracted Covid 19.”*

(P32, female, age 18–30, no pre-existing conditions, baseline)

Later in the pandemic, concerns shifted towards the removal of free testing and broader surveillance systems, alongside worries about new variants and vaccine effectiveness.

*“I am concerned about the UK government's action in ending free testing and many of the data collections and studies that may leave us vulnerable to new, more harmful variants.”*

(P35, female, age 61-70, no pre-existing condition, 24 months)

*“Also, as time passes the increased likelihood of new variants reducing the effectiveness of vaccines and resulting in more hospitalizations and deaths.”*

(P36, male, age 61-70, no pre-existing conditions, 12 months)

Workplace exposure was another recurring theme, initially among those in high-risk roles and later among those required to return to shared work environments.

*“I work in a school with children aged 13 and over who have learning disabilities, don't know how to distance, need personal care, without being allowed to wear a face covering, all the precautions that are insisted upon in other workplaces. How can I protect myself and others.”*

(P37, female, age 41–50, pre-existing condition, 3 months)

*“Being forced to return to the office in a hot desk environment with colleagues whose children keep bringing Covid home from school.”*

(P38, male, age 18-30, pre-existing condition(s), 24 months)

Policy changes reducing protections were particularly concerning at major transition points, especially for clinically vulnerable individuals.

*“As a person who is shielding, it is worrying if shielding is lifted, I do not think it is safe to go out into shops etc.”*

(P39, female, age 41-50, pre-existing condition, three months)

*“Current increasing rates of infection, hospitalisation and death - at a time when restrictions are being more or less entirely dropped. Clinically vulnerable and extremely vulnerable people are given very little extra help to keep themselves free of the virus now.”*

(P40, female, age 51–60, pre-existing condition, 24 months)

A minority also expressed concerns about the negative societal effects of increased restrictions, particularly for children and the economy.

*“I am only worried that [my children’s] education might be further disrupted if there is another wave and lockdown occurs again.”*

(P41, female, age 51-60, no pre-existing condition, three months)

*“I don't think blanket lockdowns work, just destroying the economy. If you need to shield, then do so, it's been a knee jerk hysterical reaction.”*

(P42, male, age 41–50, pre-existing conditions, 12 months)

### Government and media

Concerns about government and media centred on trust, reliability of information, and proportionality of risk communication. Concerns about government and media were often closely intertwined within participants’ accounts. For analytic purposes, comments were coded as *government concerns* when they primarily related to policy decisions, leadership, public health measures, civil liberties, or trust in government, and as *media concerns* when they focused on the quality, reliability, tone, or quantity of information and coverage. Responses that contained concerns relating to both domains could be coded into multiple categories.

At baseline, 360/11,113 (3.2%) respondents had one or more concerns about government and/or media, 235/7,048 (3.3%) at 3-months, 237/5,535 (4.2%) at 12-months, 122/4,242 (2.9%) at 18-months, and 94/3,827 (2.5%) at 24-months. Some participants viewed the government response as inadequate or poorly prepared, contributing to preventable harm.

*“That it's [COVID-19] still rife and causing terrible health and social problems and the UK government are basically just ignoring it.”*

(P44, female, age 41-50, pre-existing condition(s), 24 months)

Others felt that government measures were excessive and economically damaging.

*“Their hysteria and public opinion turning against the government was why a very sensible plan was abandoned in favour of useless draconian measures, which will be pointless as when we come out of isolation everyone will catch the bug again, we might as well get it over with rather than destroy the economy, people's careers, lose houses etc through these stupid economy harming measures, which ultimately will lead to far more deaths as with the economy depressed the NHS and social care will get less money and people will die, in far greater numbers than the COVID”*

(P45, female, age 31-40, no pre-existing conditions, baseline)

Discrepancies between official messaging and personal experiences generated anxiety and confusion.

*“The government messaging makes me feel really anxious and paranoid, but at the same time those adverts are at odds with my own experience and the experience of most of the people I know who've had it (some moderately ill).”*

(P46, female, age 51-60, no pre-existing condition, 12 months)

Trust was further undermined by perceived rule-breaking among high-profile figures and inconsistencies between UK nations, which were seen to reduce public compliance and create injustice.

*“Once there was the debacle surrounding Dominic Cummings and his arrogance regarding the regulations and spin he put on them, until then the local community had been excellent at following the guidelines (which were not hard to understand) following that and the total disregard shown by Boris [Johnson] to the rest of us. People started going out more, not socially distancing as they should.”*

(P47, female, age 61-70, no pre-existing condition, 3 months)

*“The lack of cohesion between the four nations of the UK in managing the response. This has led to significant confusion which in turn has contributed to the success of the virus in spreading.”*

(P48, male, age 61-70, pre-existing condition(s), 18 months)

Media-related concerns often focused on excessive, contradictory, or alarmist coverage that heightened anxiety.

*“Media scaremongering and contradictory information that confuses, scares and is generally unhelpful in managing the virus.”*

(P48, female, age 41-50, no pre-existing condition, 3 months)

*“It is impossible to get away from it. Even if you try. The TV will remind you of your duty.”*

(P49, female, age 71–80, pre-existing condition(s), 3 months)

Participants frequently viewed government and media concerns as interconnected, with media perceived to amplify political messaging and policy differences.

*“Too many politicians do not get the impact of the virus, they put their personal gain in terms of media presence over the health of the nation. Calling for pubs to open may get a few headlines but the deaths and illness it could cause would hit families and the economy hard.”*

(P50, male, age 61-70, no pre-existing condition, 12 months)

*“My main concern is the lack of clear information especially when England and Wales differ so much, but we hardly ever hear about Wales and their plans in the media.”*

(P51, female, age 71-80, no pre-existing condition, 3 months)

### General concerns

General concerns fell into three areas: timeline, uncertainty, and non-specific worries. Although some comments within this category were non-specific and could not be assigned to a more substantive category, the category also captured a distinct form of concern centred on uncertainty itself, including worries about the future course of the pandemic, its duration, and the possibility of returning to normal life. These types of concern decreased as the pandemic progresses, with 641/11,113 (5.8%) respondents at baseline reporting a concern in this category, 278/7,048 (3.9%) at 3-months, 218/5,535 (3.9%) at 12-months, 204/4,242 (4.8%) at 18-months, and 82/3,827 (2.1%) at 24-months. Timeline concerns focused on how long the pandemic would last and whether life would return to normal.

*“Will it ever go away or is it something we have to live with for the rest of our lives, and how our lives will be affected if a vaccine is not found.”*

(P52, female, age 61-70, pre-existing condition, 3 months)

Uncertainty about the virus, its impacts, and future developments also generated anxiety, particularly regarding unknown long-term consequences and adherence to guidance.

*“It is the fear of the unknown and anticipation of what is to come.”*

(P53, female, age 41-50, no pre-existing conditions, baseline)

Some responses expressed broader, non-specific worries about disrupted life and general health concerns that did not fit other categories, for example “*How it has ruined normal life*” (P54, female, age 71–80, pre-existing condition(s), 3 months), or “*general health concerns*” (P55, female, age 61–70, no pre-existing condition, 18 months).

## Discussion

The findings of this study highlight that concerns about COVID-19 were complex and dynamic, evolving over the course of the pandemic as people attempted to balance the need to protect themselves and others from infection, with concerns about the profound impact of the government pandemic response on daily life and society and the economy more broadly. Overall, the proportion of people reporting concerns decreased over the course of the pandemic, with concerns about personal harm being common throughout. The specific concerns reported changed over the course of the pandemic, shifting in emphasis from pro-social concerns about harm to others during the early stages to more individual-focused worries about personal harm and concerns about others not following rules during the later stages. Concerns about the social and economic impact of the pandemic also decreased over time. These findings indicate a need for ongoing holistic assessment of concerns during pandemics that extend beyond perceptions of personal harm. To fully understand the motivational context within which infection-prevention behaviours occur, conceptualisation and measurement of affective risk appraisal needs to incorporate concerns about harm to others, social and economic impacts, appropriateness and adherence with measures to control the spread of infection, and trust in government and media. A more nuanced understanding of concerns may help inform the development of adaptive, tailored public health interventions and communication strategies in future pandemics.

The findings of this study indicated that COVID-19 risk perception was underpinned by a wide range of concerns, which is consistent with previous research [39]. During the pandemic, governments largely relied on policies relating to social isolation and distancing – particularly before vaccinations were widely available – but these interventions have a considerable psychological and socio-economic cost and disproportionally affect disadvantaged populations [40]. Concerns about protection for other people were prominent in our study during the early stages of the pandemic. This may help explain previous observations of an association between COVID-19 risk perception and engagement in pro-social behaviour that has been consistently found across several countries [4].

The presence of a distinct uncertainty-focused category within the general concerns reported is consistent with the unprecedented and rapidly evolving nature of the pandemic, suggesting that uncertainty itself constituted an important source of concern for some participants independent of specific fears relating to infection, restrictions, or wider societal impacts. Previous research has noted that intolerance of uncertainty had an important influence on mental health and social isolation during the pandemic [41], indicating that this is potentially an important issue to address in pandemic response planning.

The risk of serious harm had reduced in the UK by the later stages of this study due to a combination of population immunity, widespread vaccination, and differences in the dominant virus variant, but community cases, hospitalisations and deaths still posed a significant risk to population health [42,43]. This corresponded with an overall reduction in the proportion of people in our study reporting concerns by the 24-month follow-up point, but many people remained worried. Concerns often centred about personal harm from COVID-19, compounded by perceived clinical vulnerability, worries about the impact of removal of protection measures, and a perceived decline in infection-prevention behaviour amongst members of the public. This left many of those who remained at risk from harm feeling vulnerable and forgotten by government and society more broadly.

The persistence of concerns relating to clinical vulnerability and personal harm during the later stages of the pandemic may partly reflect the demographic profile of the cohort, which included a high proportion of older adults and individuals with long-term health conditions. These groups remained at elevated risk of adverse outcomes from COVID-19 and may therefore have experienced the easing of restrictions differently from younger and healthier population groups. Exploratory descriptive analyses suggested concerns relating to personal harm were more prominent among older adults and those reporting pre-existing medical conditions, whereas concerns relating to harm to others and broader social and economic consequences were more prominent in the reports of younger participants. Concerns relating to harm to others and preventing transmission were reported more frequently among women within the cohort. Although these observations should be interpreted cautiously, as the study was not designed to make inferential comparisons between demographic groups, they suggest that different population groups may experience and prioritise pandemic-related risks in different ways. Understanding these differences may help inform more tailored public health communication and support strategies during future public health emergencies.

The overall decline in the number of people reporting concerns about COVID-19, despite the ongoing harm to public health, may have been indicative of pandemic fatigue, reflecting demotivation to follow protective behaviours that emerges gradually over time, affected by a range of emotions, experiences and perceptions [44]. However, this was also likely to have been influenced by a marked change in government policy at this time, accompanied by a shift in messaging from the need for restrictions and personal responsibility to ‘Living with COVID-19’ alongside other respiratory infections alongside other external factors such as vaccination rollout, community prevalence of COVID-19, dominant variants in circulation [45].

Previous research has highlighted the impact that low trust in government, social trust and individualistic worldviews can have on the acceptance of pandemic mitigation strategies [46,47]. However, these findings are not universal; high trust in government can potentially decrease engagement in preventative behaviour due to an increased sense of security [39,48]. Our study indicated that lack of trust in government, particularly following high-profile examples of government and associated parties breaking lockdown rules without repercussion, was particularly problematic. This impacted on people’s sense of social justice and willingness to engage with policies that were having a major impact on their civil liberties and daily lives. Perceptions of others not following rules and guidelines and lack of enforcement undermined people’s motivation and reduced their sense of control over preventing infection. Conversely, some people felt that government restrictions disproportionate given their perceived low risk of COVID-19 disease.

Concerns about others not following rules were often framed in moral terms. Responses frequently conveyed frustration, anger, and perceptions of unfairness, suggesting that concerns extended beyond infection risk alone. This may reflect broader issues relating to trust, collective responsibility, and social cohesion during a public health emergency [49,50]. For many participants, adherence to protective measures appeared to be viewed as a shared social obligation, with perceived non-compliance by others undermining confidence in collective efforts to reduce harm. These concerns may also have contributed to a reduced sense of personal control, particularly among individuals who considered themselves clinically vulnerable and perceived themselves to be dependent on the actions of others to reduce their risk of exposure.

Exposure to media can influence motivation to engage in infection-prevention behaviour and mental health outcomes, depending on perceived accuracy of the information, trust in sources of information, and type of perceived threat (health risk vs. political risk) [7,51–53]. In this study, people’s views of the government and media were intertwined, with concerns centred around a perceived discrepancy between information presented in mainstream media and people’s lived experiences, as well as perceptions that the quantity of coverage was excessive and provoked anxiety. Social media did not feature prominently in the specific concerns reported in this study, which is consistent with the quantitative findings of the COPE study which indicated that participants considered mainstream media to be more reliable than social media throughout the pandemic [6]. Our findings reinforce the importance of ensuring that communication strategies focusing on mainstream media coverage are well planned, consistent and accurate so that members of the public can have confidence in the information that they receive through these channels.

### Interpreting concerns through the lens of COM-B and PRIME Theory

The findings can be understood through the motivational constructs of COM-B and PRIME Theory. PRIME Theory would predict that motivation to engage in a behaviour involves balancing needs and impulses at a particular moment in time [12], and our findings broadly support this view. Participants’ accounts frequently reflected experiences arising from both the pandemic itself and the measures implemented to control transmission. Concerns about infection, long-term health consequences, and clinical vulnerability reflect affective processes within automatic motivation, characterised by fear, worry and anticipated harm. Concerns about transmitting infection to family members and vulnerable individuals illustrate socially oriented motives that may strengthen engagement in protective behaviours. Concerns relating to vaccination, testing, and adherence to changing guidance may also reflect reflective motivation, involving conscious evaluation and planning of protective action rather than purely automatic affective response. In contrast, concerns regarding social isolation, restrictions, economic impacts, and loss of normal activities reflect competing needs and motivations. Similarly, concerns about others not adhering to guidance may reflect perceived breakdowns in collective responsibility and social norms, potentially weakening motivation by reducing trust in the effectiveness of shared protective action.

Many of the concerns relating to social isolation, education, finances, daily activities, and mental well-being cannot be attributed solely to the direct health threat posed by SARS-CoV-2 infection. These influences were closely intertwined in people’s descriptions of their concerns and as such it is difficult to disentangle the influence of the pandemic itself from those arising from government restrictions and other external influences. The concerns reported reflect participants’ experiences of the pandemic context, highlighting how participants in this cohort experienced and balanced the combined effects of the pandemic and its societal response.

Consistent with PRIME Theory, participants frequently appeared to balance competing motives, providing insight into motivational processes that could have potentially influenced behavioural intentions over the course of the pandemic. The changing prominence of concern categories across the pandemic suggests shifts in these competing motivational processes, which were likely influenced by a wide range of internal and external factors such as habituation, fatigue, vaccination rollout, different dominant variants of SARSCoV2 in circulation, policy changes, and media messaging.

### Strengths and limitations

This study provided novel insights into the evolution of specific concerns that underpinned COVID-19 affective risk perception. An in-depth analysis of over 40,000 free-text comments from a large-scale longitudinal prospective study was carried out. As a qualitative study, the aim is to provide in-depth insights into the nature and relative prominence of concerns arising from the lived experiences of this cohort, rather than to generate population-level prevalence estimates or findings that are statistically generalisable. Nonetheless, a limitation of the study is that the COPE cohort was self-selecting and had a higher proportion of older adults, women and people with long-term conditions relative to the Welsh and UK general population, and people from ethnic minority communities were under-represented. Further, attrition over the 24-month follow-up period may have influenced the pattern of concerns observed, as participants who remained engaged in the study differed systematically from those who withdrew, with greater attrition for younger adults and those who reported that had not had a flu vaccination in the last 12 months at baseline in particular (S1 Table).

Although exploratory descriptive analyses suggested differences in the relative prominence of concern categories according to age, gender and pre-existing health conditions, these observations should be regarded as hypothesis-generating only. These findings should not be interpreted as evidence that demographic characteristics predict particular concern categories.

Exploratory subgroup analyses (S4–S6 Tables) were limited to age, gender and pre-existing medical conditions; socioeconomic background and educational attainment were not examined and represent an area for future analysis. Nonetheless, the prominence of concerns relating to personal vulnerability, severe illness and long-term health consequences may reflect the over-representation of older adults, women and people with pre-existing health conditions within the cohort. Concerns that were particularly relevant to men, younger populations, ethnic minority communities, those who are potentially less health-engaged (as assessed by self-reported flu vaccination uptake) may be under-represented. As such, we caution against assuming that the findings are representative of the wider UK population.

The free-text response question was framed to elicit people’s descriptions of their main concerns if they were worried about COVID-19. It was framed in this way to avoid assuming that respondents were worried about the pandemic, particularly given the narratives around false information and conspiracies circulating at the time [54]. However, framing the question in this way may have led to under-reporting, particularly for more minor concerns, and this should be taken into consideration when interpreting the findings. Observer bias was minimised as participants self-completed surveys without a researcher present.

Participants could report multiple concerns within a single response and therefore be coded into multiple categories simultaneously. While statistical approaches could be applied to such data, the purpose of this study was not to model longitudinal predictors or transitions between concern categories. Instead, consistent with the qualitative content analytic framework, coding frequencies were used descriptively to illustrate the relative prominence and evolution of concerns within the dataset and to support interpretation of participants’ accounts. Further, a formal inter-coder agreement statistic was not calculated, as we applied an alternative team-based quality assurance process throughout the analyses.

The findings of this study provide a rich qualitative account of the concerns raised by participants and their relative prominence within the dataset. This aligns with the purpose of the analysis, which is to understand and contextualise the lived experiences of this cohort rather than to produce statistically generalisable findings or population-level prevalence estimates. Future research is needed to develop and utilise more nuanced quantitative measures of concerns that would allow for the application of mixed-effects or Generalised Estimating Equations (GEE) approaches to examine demographic predictors of specific concern categories and longitudinal transitions between categories during pandemics. Pandemic social research planning also needs to build in robust strategies for increasing diversity in survey samples with a focus on inclusion of under-served groups.

## Conclusions

This study highlighted the dynamic and multi-dimensional nature of concerns during the COVID-19 pandemic. These findings contribute to understanding of how public concerns evolve during a prolonged public health emergency and identify a wide range of issues affecting individuals that may warrant attention in future pandemic planning. Monitoring concerns longitudinally may provide valuable insights to inform communication strategies and the development of future public health interventions.

## Supporting information

S1 Table Demographic characteristics of the COPE cohort at each survey time point. (DOCX)

S2 Table Example quotes for sub-categories at each survey time point. (XLSX)

S3 Table STROBE checklist. (DOCX)

S4 Table Mean total concerns in main categories by gender. (DOCX)

S5 Table Mean total concerns in main categories by age. (DOCX)

S6 Table Mean total concerns in main categories by pre-existing condition. (DOCX)
