# Supplementary material for: Evolving concerns: A longitudinal content analysis of free-text reports from the UK COVID-19 Public Experiences (COPE) study cohort
Source: PLoS One. 2026 Nov 10;21(11):e0348308. doi: 10.1371/journal.pone.0348308 (PMC13750560; doi:10.1371/journal.pone.0348308)
Supplement: Supplementary file 1 [file pone.0348308.s008.pdf]

1. The longitudinal design is valuable, but the potential impact of participant attrition over the five survey waves is not sufficiently addressed. It remains unclear whether those who continued participating differed from those who dropped out, which could influence the observed changes in concerns over time. A clearer assessment of retention bias would strengthen confidence in the reported trends.
2. The qualitative analysis would benefit from greater methodological clarity. Although coding procedures are described, more information on how coding consistency was maintained across time points and how disagreements were resolved is needed. This would help readers better assess the reliability and reproducibility of the findings.
3. Changes in participants' concerns are interpreted in relation to the evolving pandemic context, yet these links remain largely descriptive. Integrating key events such as lockdowns, vaccination campaigns, or policy changes more directly into the interpretation would provide a stronger explanation for the shifts observed across survey waves.
4. The findings are presented primarily at the overall cohort level, which may mask important differences between population groups. Exploring whether concerns varied according to age, health status, socioeconomic background, or other participant characteristics would enhance the practical and public health relevance of the study.
5. The sample composition limits broader generalizability. The cohort included a relatively high proportion of older adults and individuals with long-term health conditions, while some demographic groups were underrepresented. A more explicit discussion of how these characteristics may have influenced the dominant concerns identified would improve interpretation of the results.
6. While the manuscript references COM-B and PRIME Theory, the connection between the findings and these frameworks could be developed further. Clarifying how the identified concerns relate to specific theoretical constructs would strengthen the study's contribution beyond descriptive reporting.
7. The analysis focuses mainly on the prevalence of concern categories, providing a useful overview but offering limited insight into how concerns interacted in participants' lives. Greater attention to the balance between health, social, financial, and emotional concerns would provide a more nuanced understanding of the experiences and behavioral motivations underlying the reported trends.
