# Supplementary material for: Evolving concerns: A longitudinal content analysis of free-text reports from the UK COVID-19 Public Experiences (COPE) study cohort
Source: PLoS One. 2026 Nov 10;21(11):e0348308. doi: 10.1371/journal.pone.0348308 (PMC13750560; doi:10.1371/journal.pone.0348308)
Supplement: Supplementary file 2 [file pone.0348308.s001.docx]

**S1 Table: Demographic characteristics of the COPE cohort at each survey time point**

| **Variable** | **Category** | **Baseline (n=11,113)** | | **3 months (n=7,048)** | | **12 months (n=5,437)** | | **18 months (n=4,242)** | | **24 months (n=3,827)** | |
| --- | --- | --- | --- | --- | --- | --- | --- | --- | --- | --- | --- |
|  |  | **n** | **%** | **n** | **%** | **n** | **%** | **n** | **%** |  |  |
| Sex | Male | 3359 | 30.2 | 2234 | 31.7 | 1786 | 32.8 | 1450 | 34.2 | 1310 | 34.2 |
|  | Female | 7694 | 69.2 | 4781 | 67.8 | 3629 | 66.7 | 2774 | 65.4 | 2506 | 65.5 |
|  | *Missing/rather not say* | *60* | *0.6* | *20* | *0.3* | *22* | *0.4* | *18* | *0.4* | *11* | *0.4* |
| Age category | 18-30 | 810 | 7.3 | 354 | 5 | 212 | 3.9 | 128 | 3 | 93 | 2.4 |
|  | 31-40 | 1251 | 11.3 | 669 | 9.5 | 437 | 8 | 276 | 6.5 | 235 | 6.1 |
|  | 41-50 | 1459 | 13.1 | 870 | 12.3 | 628 | 11.6 | 434 | 10.2 | 393 | 10.3 |
|  | 51-60 | 2352 | 21.2 | 1483 | 21 | 1136 | 20.9 | 874 | 20.6 | 784 | 20.5 |
|  | 61-70 | 3229 | 29.1 | 2244 | 31.8 | 1865 | 34.3 | 1566 | 36.9 | 1436 | 37.5 |
|  | 71-80 | 1786 | 16.1 | 1277 | 18.1 | 1037 | 19.1 | 864 | 20.4 | 805 | 21 |
|  | 81+ | 211 | 1.9 | 146 | 2.1 | 117 | 2.2 | 97 | 2.3 | 79 | 2.1 |
|  | *Missing/rather not say* | *15* | *0.2* | *5* | *0.1* | *5* | *0.1* | *3* | *0.1* | *2* | *0* |
| Ethnicity | White (Welsh, English, Scottish, Northern Irish, British) | 10,514 | 94.6 | 6722 | 95.4 | 5174 | 95.2 | 4056 | 95.6 | 3667 | 95.8 |
|  | White other | 316 | 2.8 | 185 | 2.6 | 160 | 2.9 | 117 | 2.8 | 103 | 2.7 |
|  | Black/African/Caribbean/Black British | 14 | 0.1 | 2 | 0 | 4 | 0.1 | 2 | 0 | 1 | 0 |
|  | Asian/Asian British | 58 | 0.5 | 27 | 0.4 | 16 | 0.3 | 9 | 0.2 | 10 | 0.3 |
|  | Mixed/multiple ethnic groups | 71 | 0.6 | 42 | 0.6 | 30 | 0.6 | 19 | 0.4 | 14 | 0.4 |
|  | Other | 22 | 0.2 | 11 | 0.2 | 9 | 0.2 | 8 | 0.2 | 6 | 0.2 |
|  | *Missing/Rather not say* | *118* | *1.1* | *59* | *0.8* | *30* | *0.6* | *31* | *0.2* | *26* | *0.6* |
| **Variable** | **Category** | **Baseline (n=11,113)** | | **3 months (n=7,048)** | | **12 months (n=5,437)** | | **18 months (n=4,242)** | | **24 months (n=3,827)** | |
| Marital status | Single | 1638 | 14.7 | 912 | 12.9 | 700 | 12.9 | 512 | 12.1 | 460 | 12 |
|  | Married or in civil partnership | 6404 | 57.6 | 4249 | 60.3 | 3279 | 60.3 | 2576 | 60.7 | 2359 | 61.6 |
|  | Living with partner | 1165 | 10.5 | 661 | 9.4 | 492 | 9 | 371 | 8.7 | 314 | 8.2 |
|  | Widowed, divorced or separated | 1722 | 15.5 | 1136 | 16.1 | 893 | 16.4 | 726 | 17.1 | 643 | 16.8 |
|  | Other | 92 | 0.8 | 49 | 0.7 | 36 | 0.7 | 25 | 0.6 | 21 | 0.5 |
|  | *Missing/rather not say* | *92* | *0.8* | *41* | *0.5* | *37* | *0.7* | *32* | *0.7* | *30* | *0.8* |
| Children under 18 years living in household | Yes | 2161 | 19.4 | 1213 | 17.2 | 810 | 14.9 | 537 | 12.7 | 462 | 12.1 |
|  | No | 8886 | 80 | 5798 | 82.3 | 4599 | 84.6 | 3684 | 86.8 | 3347 | 87.5 |
|  | *Missing* | *66* | *0.6* | *37* | *0.5* | *28* | *0.5* | *21* | *0.5* | *18* | *0.5* |
| Highest level of education | No college (age 18+) education | 3418 | 30.7 | 2035 | 28.9 | 1540 | 28.3 | 1212 | 28.6 | 1094 | 28.5 |
|  | College (age 18+) education | 7458 | 67.1 | 4910 | 69.7 | 3769 | 69.3 | 2971 | 70 | 2689 | 70.2 |
|  | *Missing* | *237* | *2.1* | *103* | *1.4* | *76* | *1.4* | *59* | *1.4* | *44* | *1.2* |
| Employment status  (participants were able to select all categories that apply) | Employed full time | 3477 | 31.3 | 1987 | 28.2 | 1401 | 25.8 | 978 | 23.1 | 886 | 23.2 |
|  | Employed part time | 2124 | 19.1 | 1317 | 18.7 | 995 | 18.3 | 756 | 17.8 | 641 | 16.7 |
|  | Unemployed | 728 | 6.6 | 445 | 5.1 | 330 | 6 | 255 | 6 | 222 | 5.8 |
|  | Retired | 4560 | 41.0 | 3256 | 46.2 | 2709 | 49.8 | 2272 | 53.6 | 2101 | 54.9 |
|  | In education or training | 399 | 3.6 | 203 | 2.9 | 127 | 2.3 | 80 | 2.9 | 61 | 1.6 |
|  | *Rather not say* | *116* | *1.0* | *50* | *0.7* | *33* | *0.6* | *20* | *0.5* | *20* | *0.5* |
| Pre-existing medical condition | Yes | 5607 | 50.5 | 3623 | 51.4 | 2844 | 52.3 | 2238 | 52.8 | 2022 | 52.8 |
|  | No | 5506 | 49.5 | 3425 | 48.6 | 2593 | 47.7 | 2004 | 47.2 | 1805 | 47.2 |
| Flu vaccination in last 12 months | Yes | 6089 | 54.8 | 4074 | 57.8 | 3220 | 59.2 | 2558 | 60.3 | 2339 | 61.1 |
|  | No | 4968 | 44.7 | 2945 | 41.8 | 2195 | 40.4 | 1672 | 39.4 | 1479 | 38.6 |
|  | *Missing* | *56* | *0.5* | *29* | *0.4* | *22* | *0.4* | *12* | *0.3* | *9* | *0.2* |
