# Supplementary material for: Evolving concerns: A longitudinal content analysis of free-text reports from the UK COVID-19 Public Experiences (COPE) study cohort
Source: PLoS One. 2026 Nov 10;21(11):e0348308. doi: 10.1371/journal.pone.0348308 (PMC13750560; doi:10.1371/journal.pone.0348308)
Supplement: Supplementary file 4 [file pone.0348308.s004.docx]

**S4 Table: Concerns in main categories by gender**

|  |  |  |  |  |  | **95% CI** |  |
| --- | --- | --- | --- | --- | --- | --- | --- |
| **Main category of concern** | **Gender as binary variable (male vs. female)** | **N** | **Mean of total number of concerns reported in main category** | **Std. Deviation** | **Std. Error Mean** | **Lower** | **Upper** |
| T1 total personal harm | Male | 2405 | 0.58 | 0.691 | 0.014 | 0.01 | 0.077 |
|  | Female | 5716 | 0.54 | 0.708 | 0.009 |  |  |
| T1 total harm others | Male | 2405 | 0.4441 | 0.6532 | 0.01332 | -0.30582 | -0.235 |
|  | Female | 5716 | 0.7145 | 0.77787 | 0.01029 |  |  |
| T1 total wider social concerns | Male | 2405 | 0.28 | 0.568 | 0.012 | -0.144 | -0.08 |
|  | Female | 5716 | 0.39 | 0.715 | 0.009 |  |  |
| T1 total government and media concerns | Male | 2405 | 0.0536 | 0.22535 | 0.0046 | 0.00546 | 0.02484 |
|  | Female | 5716 | 0.0385 | 0.1933 | 0.00256 |  |  |
| T1 total concerns about preventing spread | Male | 2405 | 0.1 | 0.325 | 0.007 | -0.053 | -0.019 |
|  | Female | 5716 | 0.14 | 0.377 | 0.005 |  |  |
| T1 total general concerns | Male | 2405 | 0.08 | 0.276 | 0.006 | -0.01 | 0.016 |
|  | Female | 5716 | 0.08 | 0.275 | 0.004 |  |  |
| T2 Total personal harm | Male | 1582 | 0.72 | 0.675 | 0.017 | -0.007 | 0.076 |
|  | Female | 3708 | 0.68 | 0.725 | 0.012 |  |  |
| T2 total harm to others | Male | 1582 | 0.3 | 0.549 | 0.014 | -0.239 | -0.165 |
|  | Female | 3708 | 0.5 | 0.656 | 0.011 |  |  |
| T2 total wider social concerns | Male | 1582 | 0.19 | 0.504 | 0.013 | -0.099 | -0.032 |
|  | Female | 3708 | 0.26 | 0.585 | 0.01 |  |  |
| T2 total government and media concerns | Male | 1582 | 0.07 | 0.256 | 0.006 | 0.021 | 0.046 |
|  | Female | 3708 | 0.03 | 0.192 | 0.003 |  |  |
| T2 total prevention of spread concerns | Male | 1582 | 0.21 | 0.479 | 0.012 | -0.08 | -0.019 |
|  | Female | 3708 | 0.26 | 0.532 | 0.009 |  |  |
| T2 total general concerns | Male | 1582 | 0.06 | 0.238 | 0.006 | -0.004 | 0.023 |
|  | Female | 3708 | 0.05 | 0.223 | 0.004 |  |  |
| T3 total personal harm | Male | 1169 | 0.83 | 0.826 | 0.024 | -0.131 | -0.008 |
|  | Female | 2627 | 0.9 | 0.917 | 0.018 |  |  |
| T3 total harm to others | Male | 1169 | 0.27 | 0.57 | 0.017 | -0.276 | -0.179 |
|  | Female | 2627 | 0.5 | 0.759 | 0.015 |  |  |
| T3 total wider social concerns | Male | 1169 | 0.27 | 0.697 | 0.02 | -0.101 | -0.003 |
|  | Female | 2627 | 0.32 | 0.717 | 0.014 |  |  |
| T3 total government and media concerns | Male | 1169 | 0.07 | 0.257 | 0.008 | -0.005 | 0.028 |
|  | Female | 2627 | 0.06 | 0.234 | 0.005 |  |  |
| T3 total general concerns | Male | 1169 | 0.05 | 0.228 | 0.007 | -0.022 | 0.011 |
|  | Female | 2627 | 0.06 | 0.238 | 0.005 |  |  |
| T4 Total personal harms concerns | Male | 918 | 0.83 | 0.822 | 0.027 | -0.191 | -0.05 |
|  | Female | 1992 | 0.95 | 0.933 | 0.021 |  |  |
| T4 Total harm to others concerns | Male | 918 | 0.24 | 0.536 | 0.018 | -0.253 | -0.145 |
|  | Female | 1992 | 0.44 | 0.75 | 0.017 |  |  |
| T4 Total wider social and economic concerns | Male | 918 | 0.18 | 0.505 | 0.017 | -0.093 | -0.007 |
|  | Female | 1992 | 0.23 | 0.571 | 0.013 |  |  |
| T4 Total government and media concerns | Male | 918 | 0.07 | 0.276 | 0.009 | 0.021 | 0.055 |
|  | Female | 1992 | 0.03 | 0.181 | 0.004 |  |  |
| T4 prevention of spread concerns total | Male | 918 | 0.25 | 0.498 | 0.016 | -0.031 | 0.05 |
|  | Female | 1992 | 0.24 | 0.531 | 0.012 |  |  |
| T4 general concerns total | Male | 918 | 0.07 | 0.259 | 0.009 | -0.019 | 0.021 |
|  | Female | 1992 | 0.07 | 0.261 | 0.006 |  |  |
| T5 Personal harm concerns total | Male | 794 | 0.77 | 0.668 | 0.024 | -0.116 | 0.003 |
|  | Female | 1692 | 0.82 | 0.725 | 0.018 |  |  |
| T5 Total harm to others concerns | Male | 794 | 0.29 | 0.688 | 0.024 | -0.288 | -0.15 |
|  | Female | 1692 | 0.51 | 0.87 | 0.021 |  |  |
| T5 total concerns about wider society and economy | Male | 794 | 0.11 | 0.36 | 0.013 | -0.111 | -0.038 |
|  | Female | 1692 | 0.18 | 0.463 | 0.011 |  |  |
| T5 total concerns about government and media | Male | 794 | 0.05 | 0.231 | 0.008 | -0.001 | 0.032 |
|  | Female | 1692 | 0.03 | 0.181 | 0.004 |  |  |
| T5 Preventing Spread concerns total | Male | 794 | 0.3 | 0.57 | 0.02 | -0.049 | 0.052 |
|  | Female | 1692 | 0.3 | 0.611 | 0.015 |  |  |
| T5 General concerns total | Male | 794 | 0.03 | 0.168 | 0.006 | -0.022 | 0.01 |
|  | Female | 1692 | 0.03 | 0.193 | 0.005 |  |  |
