# Supplementary material for: Evolving concerns: A longitudinal content analysis of free-text reports from the UK COVID-19 Public Experiences (COPE) study cohort
Source: PLoS One. 2026 Nov 10;21(11):e0348308. doi: 10.1371/journal.pone.0348308 (PMC13750560; doi:10.1371/journal.pone.0348308)
Supplement: Supplementary file 5 [file pone.0348308.s005.docx]

**S5 Table: Concerns in main categories by age group**

|  |  |  |  |  |  | **95% CI** |  |
| --- | --- | --- | --- | --- | --- | --- | --- |
| **Main category** | **Age as a binary variable, up to 60 vs. 61 or over** | **N** | **Mean of total number of concerns reported in main category** | **Std. Deviation** | **Std. Error Mean** | **Lower** | **Upper** |
| T1 total personal harm | 60 or under | 4386 | 0.49 | 0.701 | 0.011 | -0.173 | -0.113 |
|  | 61 or over | 3769 | 0.63 | 0.698 | 0.011 |  |  |
| T1 total harm others | 60 or under | 4386 | 0.7937 | 0.78705 | 0.01188 | 0.31549 | 0.37929 |
|  | 61 or over | 3769 | 0.4463 | 0.66361 | 0.01081 |  |  |
| T1 total wider social concerns | 60 or under | 4386 | 0.44 | 0.769 | 0.012 | 0.151 | 0.21 |
|  | 61 or over | 3769 | 0.26 | 0.531 | 0.009 |  |  |
| T1 total government and media concerns | 60 or under | 4386 | 0.0511 | 0.2212 | 0.00334 | 0.00797 | 0.02572 |
|  | 61 or over | 3769 | 0.0342 | 0.18183 | 0.00296 |  |  |
| T1 total concerns about preventing spread | 60 or under | 4386 | 0.14 | 0.377 | 0.006 | 0.005 | 0.036 |
|  | 61 or over | 3769 | 0.12 | 0.345 | 0.006 |  |  |
| T1 total general concerns | 60 or under | 4386 | 0.08 | 0.279 | 0.004 | -0.012 | 0.012 |
|  | 61 or over | 3769 | 0.08 | 0.273 | 0.004 |  |  |
| T2 Total personal harm | 60 or under | 2491 | 0.61 | 0.709 | 0.014 | -0.188 | -0.112 |
|  | 61 or over | 2823 | 0.76 | 0.706 | 0.013 |  |  |
| T2 total harm to others | 60 or under | 2491 | 0.59 | 0.675 | 0.014 | 0.239 | 0.306 |
|  | 61 or over | 2823 | 0.31 | 0.564 | 0.011 |  |  |
| T2 total wider social concerns | 60 or under | 2491 | 0.27 | 0.613 | 0.012 | 0.024 | 0.085 |
|  | 61 or over | 2823 | 0.21 | 0.518 | 0.01 |  |  |
| T2 total government and media concerns | 60 or under | 2491 | 0.04 | 0.208 | 0.004 | -0.019 | 0.004 |
|  | 61 or over | 2823 | 0.05 | 0.22 | 0.004 |  |  |
| T2 total prevention of spread concerns | 60 or under | 2491 | 0.24 | 0.531 | 0.011 | -0.025 | 0.031 |
|  | 61 or over | 2823 | 0.24 | 0.505 | 0.009 |  |  |
| T2 total general concerns | 60 or under | 2491 | 0.05 | 0.223 | 0.004 | -0.021 | 0.004 |
|  | 61 or over | 2823 | 0.06 | 0.232 | 0.004 |  |  |
| T3 total personal harm | 60 or under | 1678 | 0.87 | 0.888 | 0.022 | -0.077 | 0.037 |
|  | 61 or over | 2132 | 0.89 | 0.891 | 0.019 |  |  |
| T3 total harm to others | 60 or under | 1678 | 0.57 | 0.782 | 0.019 | 0.215 | 0.305 |
|  | 61 or over | 2132 | 0.31 | 0.632 | 0.014 |  |  |
| T3 total wider social concerns | 60 or under | 1678 | 0.35 | 0.784 | 0.019 | 0.029 | 0.12 |
|  | 61 or over | 2132 | 0.27 | 0.65 | 0.014 |  |  |
| T3 total government and media concerns | 60 or under | 1678 | 0.07 | 0.256 | 0.006 | -0.001 | 0.03 |
|  | 61 or over | 2132 | 0.05 | 0.23 | 0.005 |  |  |
| T3 total general concerns | 60 or under | 1678 | 0.05 | 0.223 | 0.005 | -0.024 | 0.007 |
|  | 61 or over | 2132 | 0.06 | 0.245 | 0.005 |  |  |
| T4 Total personal harms concerns | 60 or under | 1187 | 0.87 | 0.902 | 0.026 | -0.136 | -0.003 |
|  | 61 or over | 1737 | 0.94 | 0.906 | 0.022 |  |  |
| T4 Total harm to others concerns | 60 or under | 1187 | 0.53 | 0.789 | 0.023 | 0.205 | 0.306 |
|  | 61 or over | 1737 | 0.27 | 0.602 | 0.014 |  |  |
| T4 Total wider social and economic concerns | 60 or under | 1187 | 0.26 | 0.628 | 0.018 | 0.026 | 0.108 |
|  | 61 or over | 1737 | 0.19 | 0.491 | 0.012 |  |  |
| T4 Total government and media concerns | 60 or under | 1187 | 0.04 | 0.202 | 0.006 | -0.023 | 0.009 |
|  | 61 or over | 1737 | 0.04 | 0.225 | 0.005 |  |  |
| T4 prevention of spread concerns total | 60 or under | 1187 | 0.24 | 0.53 | 0.015 | -0.05 | 0.028 |
|  | 61 or over | 1737 | 0.25 | 0.517 | 0.012 |  |  |
| T4 general concerns total | 60 or under | 1187 | 0.07 | 0.255 | 0.007 | -0.026 | 0.012 |
|  | 61 or over | 1737 | 0.07 | 0.266 | 0.006 |  |  |
| T5 Personal harm concerns total | 60 or under | 973 | 0.76 | 0.726 | 0.023 | -0.133 | -0.019 |
|  | 61 or over | 1522 | 0.84 | 0.696 | 0.018 |  |  |
| T5 Total harm to others concerns | 60 or under | 973 | 0.61 | 0.93 | 0.03 | 0.22 | 0.351 |
|  | 61 or over | 1522 | 0.33 | 0.723 | 0.019 |  |  |
| T5 total concerns about wider society and economy | 60 or under | 973 | 0.19 | 0.49 | 0.016 | 0.013 | 0.083 |
|  | 61 or over | 1522 | 0.14 | 0.394 | 0.01 |  |  |
| T5 total concerns about government and media | 60 or under | 973 | 0.05 | 0.224 | 0.007 | -0.002 | 0.03 |
|  | 61 or over | 1522 | 0.03 | 0.18 | 0.005 |  |  |
| T5 Preventing Spread concerns total | 60 or under | 973 | 0.29 | 0.601 | 0.019 | -0.056 | 0.041 |
|  | 61 or over | 1522 | 0.3 | 0.596 | 0.015 |  |  |
| T5 General concerns total | 60 or under | 973 | 0.03 | 0.187 | 0.006 | -0.017 | 0.013 |
|  | 61 or over | 1522 | 0.03 | 0.184 | 0.005 |  |  |
