# Supplementary material for: Evolving concerns: A longitudinal content analysis of free-text reports from the UK COVID-19 Public Experiences (COPE) study cohort
Source: PLoS One. 2026 Nov 10;21(11):e0348308. doi: 10.1371/journal.pone.0348308 (PMC13750560; doi:10.1371/journal.pone.0348308)
Supplement: Supplementary file 6 [file pone.0348308.s006.docx]

**S6 Table: Concerns in main categories by report of pre-existing condition(s) at baseline**

|  |  |  |  |  |  | **95% CI** |  |
| --- | --- | --- | --- | --- | --- | --- | --- |
| **Main category of concern** | **Pre-existing medical condition at baseline, none vs. one or more conditions** | **N** | **Mean of total number of concerns reported in main category** | **Std. Deviation** | **Std. Error Mean** | **Lower** | **Upper** |
| T1 total personal harm | No | 3998 | 0.42 | 0.626 | 0.01 | -0.301 | -0.241 |
|  | Yes | 4164 | 0.69 | 0.746 | 0.012 |  |  |
| T1 total harm others | No | 3998 | 0.7554 | 0.77727 | 0.01229 | 0.208 | 0.2725 |
|  | Yes | 4164 | 0.5151 | 0.70856 | 0.01098 |  |  |
| T1 total wider social concerns | No | 3998 | 0.42 | 0.724 | 0.011 | 0.085 | 0.143 |
|  | Yes | 4164 | 0.3 | 0.622 | 0.01 |  |  |
| T1 total government and media concerns | No | 3998 | 0.0455 | 0.20967 | 0.00332 | -0.0044 | 0.01331 |
|  | Yes | 4164 | 0.0411 | 0.19847 | 0.00308 |  |  |
| T1 total concerns about preventing spread | No | 3998 | 0.13 | 0.369 | 0.006 | -0.01 | 0.022 |
|  | Yes | 4164 | 0.13 | 0.357 | 0.006 |  |  |
| T1 total general concerns | No | 3998 | 0.09 | 0.292 | 0.005 | 0.006 | 0.03 |
|  | Yes | 4164 | 0.07 | 0.26 | 0.004 |  |  |
| T2 Total personal harm | No | 2447 | 0.55 | 0.664 | 0.013 | -0.292 | -0.217 |
|  | Yes | 2869 | 0.81 | 0.729 | 0.014 |  |  |
| T2 total harm to others | No | 2447 | 0.54 | 0.672 | 0.014 | 0.154 | 0.222 |
|  | Yes | 2869 | 0.36 | 0.585 | 0.011 |  |  |
| T2 total wider social concerns | No | 2447 | 0.27 | 0.592 | 0.012 | 0.024 | 0.085 |
|  | Yes | 2869 | 0.21 | 0.54 | 0.01 |  |  |
| T2 total government and media concerns | No | 2447 | 0.04 | 0.204 | 0.004 | -0.016 | 0.007 |
|  | Yes | 2869 | 0.05 | 0.223 | 0.004 |  |  |
| T2 total prevention of spread concerns | No | 2447 | 0.24 | 0.524 | 0.011 | -0.024 | 0.032 |
|  | Yes | 2869 | 0.24 | 0.511 | 0.01 |  |  |
| T2 total general concerns | No | 2447 | 0.06 | 0.245 | 0.005 | 0.001 | 0.026 |
|  | Yes | 2869 | 0.05 | 0.211 | 0.004 |  |  |
| T3 total personal harm | No | 1739 | 0.79 | 0.875 | 0.021 | -0.216 | -0.103 |
|  | Yes | 2073 | 0.95 | 0.895 | 0.02 |  |  |
| T3 total harm to others | No | 1739 | 0.53 | 0.751 | 0.018 | 0.138 | 0.229 |
|  | Yes | 2073 | 0.34 | 0.669 | 0.015 |  |  |
| T3 total wider social concerns | No | 1739 | 0.37 | 0.807 | 0.019 | 0.079 | 0.17 |
|  | Yes | 2073 | 0.25 | 0.623 | 0.014 |  |  |
| T3 total government and media concerns | No | 1739 | 0.06 | 0.247 | 0.006 | -0.013 | 0.018 |
|  | Yes | 2073 | 0.06 | 0.238 | 0.005 |  |  |
| T3 total general concerns | No | 1739 | 0.07 | 0.255 | 0.006 | 0.006 | 0.036 |
|  | Yes | 2073 | 0.05 | 0.218 | 0.005 |  |  |
| T4 Total personal harms concerns | No | 1320 | 0.77 | 0.827 | 0.023 | -0.333 | -0.202 |
|  | Yes | 1606 | 1.03 | 0.948 | 0.024 |  |  |
| T4 Total harm to others concerns | No | 1320 | 0.42 | 0.718 | 0.02 | 0.029 | 0.13 |
|  | Yes | 1606 | 0.34 | 0.674 | 0.017 |  |  |
| T4 Total wider social and economic concerns | No | 1320 | 0.26 | 0.595 | 0.016 | 0.032 | 0.112 |
|  | Yes | 1606 | 0.19 | 0.511 | 0.013 |  |  |
| T4 Total government and media concerns | No | 1320 | 0.04 | 0.218 | 0.006 | -0.013 | 0.018 |
|  | Yes | 1606 | 0.04 | 0.215 | 0.005 |  |  |
| T4 prevention of spread concerns total | No | 1320 | 0.26 | 0.535 | 0.015 | -0.026 | 0.051 |
|  | Yes | 1606 | 0.24 | 0.512 | 0.013 |  |  |
| T4 general concerns total | No | 1320 | 0.08 | 0.276 | 0.008 | -0.005 | 0.033 |
|  | Yes | 1606 | 0.06 | 0.249 | 0.006 |  |  |
| T5 Personal harm concerns total | No | 1103 | 0.7 | 0.701 | 0.021 | -0.25 | -0.139 |
|  | Yes | 1393 | 0.89 | 0.703 | 0.019 |  |  |
| T5 Total harm to others concerns | No | 1103 | 0.53 | 0.89 | 0.027 | 0.107 | 0.236 |
|  | Yes | 1393 | 0.36 | 0.755 | 0.02 |  |  |
| T5 total concerns about wider society and economy | No | 1103 | 0.19 | 0.473 | 0.014 | 0.022 | 0.091 |
|  | Yes | 1393 | 0.13 | 0.4 | 0.011 |  |  |
| T5 total concerns about government and media | No | 1103 | 0.04 | 0.211 | 0.006 | -0.007 | 0.025 |
|  | Yes | 1393 | 0.03 | 0.188 | 0.005 |  |  |
| T5 Preventing Spread concerns total | No | 1103 | 0.31 | 0.591 | 0.018 | -0.026 | 0.068 |
|  | Yes | 1393 | 0.29 | 0.603 | 0.016 |  |  |
| T5 General concerns total | No | 1103 | 0.04 | 0.209 | 0.006 | 0.001 | 0.03 |
|  | Yes | 1393 | 0.03 | 0.163 | 0.004 |  |  |
