# Supplementary material for: Evolving concerns: A longitudinal content analysis of free-text reports from the UK COVID-19 Public Experiences (COPE) study cohort
Source: PLoS One. 2026 Nov 10;21(11):e0348308. doi: 10.1371/journal.pone.0348308 (PMC13750560; doi:10.1371/journal.pone.0348308)
Supplement: Supplementary file 7 [file pone.0348308.s007.docx]

**Evolving concerns about the COVID-19 pandemic: A content analysis of free-text reports from the UK COVID-19 Public Experiences (COPE) study cohort over a two-year period**

**Title**

Long and may benefit from simplification.

The phrase “over a two-year period” is informative but somewhat repetitive given the longitudinal context already implied.

Consider a more concise title while preserving the key methodological aspect and longitudinal emphasis.

**Abstract**

The abstract is well structured and provides a concise overview of objectives, methods, results, and conclusions. However, a numerical inconsistency requires correction. The abstract reports **41,564** coded responses, whereas the Results section later reports **41,568** comments. This discrepancy should be resolved.

The conclusion section may slightly overstate implications by implying direct effects on future behavioral interventions. Since the analysis focuses on concerns rather than measured behavior, wording should remain more cautious.

**Keywords**

Please use keywords from the Mesh.

**Introduction**

The introduction establishes the rationale for studying changing COVID-19 concerns and appropriately distinguishes affective risk perception from broader risk constructs. The literature review is generally comprehensive and logically structured.

However, several issues require attention.

The opening sentence contains a grammatical issue:

"During pandemics perception of risk can strongly influenced people’s engagement..."

This should be revised for clarity and language accuracy.

The introduction strongly argues that evolving concerns influence infection-prevention behavior, yet the manuscript does not directly measure behavior in the current analysis. The distinction between concern and behavior should be clarified earlier to avoid creating expectations that cannot be addressed by the study design.

The justification for the study would also benefit from a clearer explanation of why existing quantitative longitudinal studies were insufficient and why qualitative content analysis specifically offers additional value.

**Method**

**The COVID-19 Public Experiences (COPE) Study**

The longitudinal design is a notable strength and allows examination of changes over time.

However, substantial participant attrition occurred across survey waves, declining from 11,113 participants at baseline to 3,827 participants at 24 months.

The manuscript does not sufficiently discuss the implications of this reduction. Attrition bias may have substantially influenced the nature of concerns expressed at later time points. Participants remaining in a two-year longitudinal study may systematically differ in motivation, health status, or anxiety profile from those who withdrew.

The authors should discuss this limitation more critically and consider whether baseline comparisons between retained and non-retained participants were performed.

**Theoretical approach**

The manuscript introduces COM-B and PRIME theory as conceptual frameworks guiding interpretation.

Although theoretically relevant, the integration appears relatively superficial throughout the manuscript. The theories are discussed in considerable detail initially, but subsequent analysis remains largely descriptive.

The manuscript would be strengthened by demonstrating how individual concern categories align with theoretical constructs. For example: Which concerns correspond to automatic versus reflective motivation? How do competing needs proposed within PRIME theory emerge within the data? Which findings support specific theoretical mechanisms?

**Study population and recruitment**

The authors describe recruitment procedures adequately and provide demographic information.

However, representativeness remains a major concern.

The study population consisted predominantly of: Female participants (69.2%), White British participants (95.8%) and Older adults (>51 years: 68.3%).

COVID-19 concerns differed substantially across demographic groups throughout the pandemic. Therefore, the lack of diversity limits broader transferability.

The discussion should more critically acknowledge the possibility that the dominant concern patterns identified may largely reflect this specific demographic profile.

**Materials and measures**

The open-ended survey question enabled broad capture of participant experiences and concerns.

However, the wording:

"If you are worried about coronavirus (COVID-19), please tell us what your main concerns are"

may unintentionally introduce selection bias.

Participants not identifying themselves as “worried” may provide shorter responses or omit concerns entirely. The framing itself could influence the type and intensity of responses generated.

The authors should discuss whether this wording may have shaped findings.

**Analysis**

The qualitative content analysis approach is appropriate given the volume of responses.

However, several methodological issues require clarification.

The coding framework was developed from baseline data and then applied deductively to later surveys.

Although practical, this strategy risks anchoring later analyses to concerns dominant during the earliest pandemic stage. Novel issues emerging later may have been under-recognized.

Further discussion of safeguards against framework rigidity would strengthen confidence in findings.

Additionally, although a 10% sample underwent verification, no formal inter-coder agreement statistics are reported.

Given involvement of eleven coders, further information regarding coding consistency would improve methodological transparency.

The rationale for reporting detailed coder demographics should also be clarified. If reflexivity was intended, this should be explicitly stated.

**Results**

**Personal harm from COVID-19**

This section clearly describes temporal changes in concern categories.

However, frequency of concerns should not necessarily be interpreted as equivalent to psychological importance or intensity. Less frequently reported concerns may still carry substantial emotional significance.

Clarification regarding interpretation of frequency data would strengthen the analysis.

Several participant quotations are useful, although some are relatively long and occasionally interrupt narrative flow. More concise quotations may improve readability.

**Harm to others from COVID-19**

The findings are well presented and demonstrate an interesting shift from collective concerns toward more individual concerns over time.

However, interpretation should remain cautious regarding causal inference. Changes in concern patterns may reflect numerous external influences including vaccination rollout, policy changes, and media messaging.

These contextual factors should be more explicitly acknowledged.

**Social and economic impacts**

This section highlights important competing concerns beyond infection risk.

The findings strongly support the notion that public concerns extended beyond health outcomes.

However, it remains difficult to separate effects arising from pandemic severity versus governmental restriction measures. More explicit discussion distinguishing these factors would strengthen interpretation.

**Preventing spread of COVID-19**

The section presents important observations regarding perceived rule adherence and public frustration.

The moral framing of non-compliance is particularly interesting and may warrant deeper interpretation.

The authors may also consider whether concern about others' behavior reflects broader concepts such as trust, collective responsibility, or perceived social cohesion.

**Government and media**

This section captures a notable range of views.

However, government and media concerns often overlap substantially. Participants frequently discuss both simultaneously.

Further explanation regarding how these categories were differentiated analytically would improve clarity.

**General concerns**

The findings are clearly presented.

However, “general concerns” may partially function as a residual category rather than a distinct conceptual category.

The authors should clarify whether this category represented a meaningful phenomenon or simply uncategorized responses.

**Discussion**

The discussion appropriately summarizes key findings and links them to prior literature.

However, several interpretations extend beyond direct evidence.

The manuscript occasionally implies explanatory relationships between concerns and infection-prevention behavior despite behavior not being directly examined in this qualitative dataset.

Interpretation should remain aligned with the study design.

The discussion would also benefit from greater attention to: Attrition bias, Limited population diversity, Coding limitations, Potential response bias, Frequency versus psychological importance

These limitations deserve more detailed treatment.

**Conclusion**

The conclusion generally reflects the results.

However, statements suggesting direct implications for adaptive public health interventions may be somewhat stronger than warranted based on the available evidence.

More cautious wording emphasizing hypothesis generation and informing future work may be preferable.
