# Supplementary material for: Evolving concerns: A longitudinal content analysis of free-text reports from the UK COVID-19 Public Experiences (COPE) study cohort
Source: PLoS One. 2026 Nov 10;21(11):e0348308. doi: 10.1371/journal.pone.0348308 (PMC13750560; doi:10.1371/journal.pone.0348308)
Supplement: Supplementary file 8 [file pone.0348308.s009.docx]

Dear Professor Kolahi,

Thank you for the opportunity to revise our manuscript ‘Evolving concerns: A longitudinal content analysis of free-text reports from the UK COVID-19 Public Experiences (COPE) study cohort’ (PONE-D-26-18243R1).

We have responded to the comments from the editor as follows:

1. Please clarify that the observed changes in concern frequencies represent differences between survey waves and do not necessarily demonstrate changes within individual participants, particularly given the substantial attrition.

We have amended the text in the analysis section on page 11 (lines 258-259) to explicitly clarify this: ‘These analyses were intended to provide contextual insight rather than formal subgroup comparisons, highlighting differences in concerns reported at each survey wave overall as opposed to changes at an individual level.’

2. Please ensure that the abstract and results consistently distinguish between the number of coded responses, the number of individual participants, and the frequency of concern categories, as participants could report multiple concerns.

The abstract has been amended to ensure that it is clear that respondents could report multiple concerns, and to clearly indicate where findings relate to frequency of concerns within a category or number of respondents reporting concerns within each category. We have edited the abstract to ensure that it remains within the 300-word limit. We have also reviewed and updated the results section to ensure that text and tables clearly indicate where frequency of concerns vs. number of respondents is indicated. These amendments have been marked using track changes in the abstract and results sections of the manuscript.

3. Please revise the statement in the Introduction suggesting that the study examines changes in concerns at a population level. The non-representative sample and selective attrition do not support population-level generalization.

Thank you for this observation. We have revised the Introduction to remove references to ‘population-level’ shifts in concerns and instead describe the study as examining changes in concerns reported within a large longitudinal community cohort.

4. Please clarify that the absence of mutually exclusive concern categories does not, by itself, preclude longitudinal statistical analysis. The decision to retain a descriptive qualitative approach should instead be justified by the study objectives and analytical framework.

We have revised the manuscript to clarify that our decision to adopt a descriptive qualitative approach was driven by the study aims and content analysis approach, which sought to characterise the nature and evolution of concerns expressed by participants, rather than to model predictors or transitions between concern categories. We have amended the Analysis and Strengths and Limitations sections to reflect this.

5. Please ensure that the exploratory demographic comparisons are consistently described as descriptive findings rather than evidence of demographic predictors of concern categories.

Thank you for this comment. We have reviewed the manuscript to ensure that we consistently describe these findings as exploratory descriptive patterns observed within the cohort, and have avoided language implying prediction, association, or causal inference. Additional clarification has been added to the Analysis, Discussion, and Strengths and Limitations sections to emphasise that these findings are hypothesis-generating only.

6. Please correct the inconsistency in the corresponding author's email address between the manuscript title page and the Data Availability Statement.

Many thanks for highlighting this, we have corrected the error in the Data Availability Statement.

Yours faithfully,

Dr Rhiannon Phillips
