# Supplementary material for: Evolving concerns: A longitudinal content analysis of free-text reports from the UK COVID-19 Public Experiences (COPE) study cohort
Source: PLoS One. 2026 Nov 10;21(11):e0348308. doi: 10.1371/journal.pone.0348308 (PMC13750560; doi:10.1371/journal.pone.0348308)
Supplement: Peer review history

# Peer review: Decision Letter 0

**DOI:** 10.1371/journal.pone.0348308.r001

**Authors:** Jafar Kolahi

6 Jul 2026

Dear Dr. Phillips,

Thank you for submitting your manuscript to PLOS ONE. After careful consideration, we feel that it has merit but does not fully meet PLOS ONE’s publication criteria as it currently stands. Therefore, we invite you to submit a revised version of the manuscript that addresses the points raised during the review process.

Please submit your revised manuscript by Aug 20 2026 11:59PM. If you will need more time than this to complete your revisions, please reply to this message or contact the journal office at plosone@plos.org. When you're ready to submit your revision, log on to https://www.editorialmanager.com/pone/ and select the 'Submissions Needing Revision' folder to locate your manuscript file.

- A letter that responds to each point raised by the academic editor and reviewer(s). You should upload this letter as a separate file labeled 'Response to Reviewers'.
- A marked-up copy of your manuscript that highlights changes made to the original version. You should upload this as a separate file labeled 'Revised Manuscript with Track Changes'.
- An unmarked version of your revised paper without tracked changes. You should upload this as a separate file labeled 'Manuscript'.

If you would like to make changes to your financial disclosure, please include your updated statement in your cover letter. Guidelines for resubmitting your figure files are available below the reviewer comments at the end of this letter.

If applicable, we recommend that you deposit your laboratory protocols in protocols.io to enhance the reproducibility of your results. Protocols.io assigns your protocol its own identifier (DOI) so that it can be cited independently in the future. For instructions see: https://journals.plos.org/plosone/s/submission-guidelines#loc-laboratory-protocols. Additionally, PLOS ONE offers an option for publishing peer-reviewed Lab Protocol articles, which describe protocols hosted on protocols.io. Read more information on sharing protocols at https://plos.org/protocols?utm_medium=editorial-email&utm_source=authorletters&utm_campaign=protocols.

As the corresponding author, your ORCID iD is verified in the submission system and will appear in the published article. PLOS supports the use of ORCID, and we encourage all coauthors to register for an ORCID iD and use it as well. Please encourage your coauthors to verify their ORCID iD within the submission system before final acceptance, as unverified ORCID iDs will not appear in the published article. *Only*  the individual author can complete the verification step; PLOS staff *cannot*  verify ORCID iDs on behalf of authors.

We look forward to receiving your revised manuscript.

Kind regards,

Jafar Kolahi

Academic Editor

PLOS One

Journal Requirements:

When submitting your revision, we need you to address these additional requirements.

1. Please ensure that your manuscript meets PLOS ONE's style requirements, including those for file naming. The PLOS ONE style templates can be found at https://journals.plos.org/plosone/s/file?id=wjVg/PLOSOne_formatting_sample_main_body.pdf and https://journals.plos.org/plosone/s/file?id=ba62/PLOSOne_formatting_sample_title_authors_affiliations.pdf

2. We note that the grant information you provided in the ‘Funding Information’ and ‘Financial Disclosure’ sections do not match.

When you resubmit, please ensure that you provide the correct grant numbers for the awards you received for your study in the ‘Funding Information’ section.

3. Thank you for stating the following financial disclosure:

[Sêr Cymru III Tackling COVID-19 grant (Project number WG 90), Cardiff Metropolitan University 'Get Started' award (internal funding), Cardiff University Division of Population Medicine internal funding.].

Please state what role the funders took in the study.  If the funders had no role, please state: "The funders had no role in study design, data collection and analysis, decision to publish, or preparation of the manuscript."

If this statement is not correct you must amend it as needed.

Please include this amended Role of Funder statement in your cover letter; we will change the online submission form on your behalf.

4. Please remove any funding-related text from the manuscript. Please note that funding information should not appear in any section or other areas of your manuscript. We will publish the funding statement that is present in the Financial Disclosure section of the online submission form.

5. Thank you for stating the following in your Competing Interests section:

[No authors have competing interests].

Please complete your Competing Interests on the online submission form to state any Competing Interests. If you have no competing interests, please state "The authors have declared that no competing interests exist.", as detailed online in our guide for authors at http://journals.plos.org/plosone/s/submit-now

This information should be included in your cover letter; we will change the online submission form on your behalf.

6. For studies involving third-party data, we encourage authors to share any data specific to their analyses that they can legally distribute. PLOS recognizes, however, that authors may be using third-party data they do not have the rights to share. When third-party data cannot be publicly shared, authors must provide all information necessary for interested researchers to apply to gain access to the data. (https://journals.plos.org/plosone/s/data-availability#loc-acceptable-data-access-restrictions)

For any third-party data that the authors cannot legally distribute, they should include the following information in their Data Availability Statement upon submission:

1) A description of the data set and the third-party source

2) If applicable, verification of permission to use the data set

3) Confirmation of whether the authors received any special privileges in accessing the data that other researchers would not have

4) All necessary contact information others would need to apply to gain access to the data

If the reviewer comments include a recommendation to cite specific previously published works, please review and evaluate these publications to determine whether they are relevant and should be cited. There is no requirement to cite these works unless the editor has indicated otherwise.

Please review your reference list to ensure that it is complete and correct. If you have cited papers that have been retracted, please include the rationale for doing so in the manuscript text, or remove these references and replace them with relevant current references. Any changes to the reference list should be mentioned in the rebuttal letter that accompanies your revised manuscript. If you need to cite a retracted article, indicate the article’s retracted status in the References list and also include a citation and full reference for the retraction notice.

Additional Editor Comments:

The manuscript primarily presents descriptive frequencies of concern categories across survey waves. To strengthen the quantitative component, the authors should consider formally evaluating temporal changes using longitudinal statistical methods, such as mixed-effects logistic regression or generalized estimating equations (GEE), which appropriately account for repeated measurements within participants. In addition, the authors may consider examining whether demographic and clinical characteristics (e.g., age, sex, education, and pre-existing medical conditions) are associated with the likelihood of reporting specific concern categories. Such analyses would provide valuable insights into population subgroups at greater risk of particular concerns and enhance the practical implications of the findings.

Consider adding a Sankey (alluvial) diagram illustrating transitions in participants' primary concern categories across survey waves. Such a figure would provide an intuitive visualization of how concerns evolved over time.

[Note: HTML markup is below. Please do not edit.]

Reviewers' comments:

Reviewer's Responses to Questions

**Comments to the Author**

1. Is the manuscript technically sound, and do the data support the conclusions?

Reviewer #1: Partly

Reviewer #2: Yes

**********

2. Has the statistical analysis been performed appropriately and rigorously?

Reviewer #1: No

Reviewer #2: Yes

**********

3. Have the authors made all data underlying the findings in their manuscript fully available?

The PLOS Data policy

Reviewer #1: Yes

Reviewer #2: Yes

**********

4. Is the manuscript presented in an intelligible fashion and written in standard English?

Reviewer #1: Yes

Reviewer #2: Yes

**********

Reviewer #1: Title

Long and may benefit from simplification.

The phrase “over a two-year period” is informative but somewhat repetitive given the longitudinal context already implied.

Consider a more concise title while preserving the key methodological aspect and longitudinal emphasis.

Abstract

The abstract is well structured and provides a concise overview of objectives, methods, results, and conclusions. However, a numerical inconsistency requires correction. The abstract reports 41,564 coded responses, whereas the Results section later reports 41,568 comments. This discrepancy should be resolved.

The conclusion section may slightly overstate implications by implying direct effects on future behavioral interventions. Since the analysis focuses on concerns rather than measured behavior, wording should remain more cautious.

Keywords

Please use keywords from the Mesh.

Introduction

The introduction establishes the rationale for studying changing COVID-19 concerns and appropriately distinguishes affective risk perception from broader risk constructs. The literature review is generally comprehensive and logically structured.

However, several issues require attention.

The opening sentence contains a grammatical issue:

"During pandemics perception of risk can strongly influenced people’s engagement..."

This should be revised for clarity and language accuracy.

The introduction strongly argues that evolving concerns influence infection-prevention behavior, yet the manuscript does not directly measure behavior in the current analysis. The distinction between concern and behavior should be clarified earlier to avoid creating expectations that cannot be addressed by the study design.

The justification for the study would also benefit from a clearer explanation of why existing quantitative longitudinal studies were insufficient and why qualitative content analysis specifically offers additional value.

Method

The COVID-19 Public Experiences (COPE) Study

The longitudinal design is a notable strength and allows examination of changes over time.

However, substantial participant attrition occurred across survey waves, declining from 11,113 participants at baseline to 3,827 participants at 24 months.

The manuscript does not sufficiently discuss the implications of this reduction. Attrition bias may have substantially influenced the nature of concerns expressed at later time points. Participants remaining in a two-year longitudinal study may systematically differ in motivation, health status, or anxiety profile from those who withdrew.

The authors should discuss this limitation more critically and consider whether baseline comparisons between retained and non-retained participants were performed.

Theoretical approach

The manuscript introduces COM-B and PRIME theory as conceptual frameworks guiding interpretation.

Although theoretically relevant, the integration appears relatively superficial throughout the manuscript. The theories are discussed in considerable detail initially, but subsequent analysis remains largely descriptive.

The manuscript would be strengthened by demonstrating how individual concern categories align with theoretical constructs. For example: Which concerns correspond to automatic versus reflective motivation? How do competing needs proposed within PRIME theory emerge within the data? Which findings support specific theoretical mechanisms?

Study population and recruitment

The authors describe recruitment procedures adequately and provide demographic information.

However, representativeness remains a major concern.

The study population consisted predominantly of: Female participants (69.2%), White British participants (95.8%) and Older adults (>51 years: 68.3%).

COVID-19 concerns differed substantially across demographic groups throughout the pandemic. Therefore, the lack of diversity limits broader transferability.

The discussion should more critically acknowledge the possibility that the dominant concern patterns identified may largely reflect this specific demographic profile.

Materials and measures

The open-ended survey question enabled broad capture of participant experiences and concerns.

However, the wording:

"If you are worried about coronavirus (COVID-19), please tell us what your main concerns are"

may unintentionally introduce selection bias.

Participants not identifying themselves as “worried” may provide shorter responses or omit concerns entirely. The framing itself could influence the type and intensity of responses generated.

The authors should discuss whether this wording may have shaped findings.

Analysis

The qualitative content analysis approach is appropriate given the volume of responses.

However, several methodological issues require clarification.

The coding framework was developed from baseline data and then applied deductively to later surveys.

Although practical, this strategy risks anchoring later analyses to concerns dominant during the earliest pandemic stage. Novel issues emerging later may have been under-recognized.

Further discussion of safeguards against framework rigidity would strengthen confidence in findings.

Additionally, although a 10% sample underwent verification, no formal inter-coder agreement statistics are reported.

Given involvement of eleven coders, further information regarding coding consistency would improve methodological transparency.

The rationale for reporting detailed coder demographics should also be clarified. If reflexivity was intended, this should be explicitly stated.

Results

Personal harm from COVID-19

This section clearly describes temporal changes in concern categories.

However, frequency of concerns should not necessarily be interpreted as equivalent to psychological importance or intensity. Less frequently reported concerns may still carry substantial emotional significance.

Clarification regarding interpretation of frequency data would strengthen the analysis.

Several participant quotations are useful, although some are relatively long and occasionally interrupt narrative flow. More concise quotations may improve readability.

Harm to others from COVID-19

The findings are well presented and demonstrate an interesting shift from collective concerns toward more individual concerns over time.

However, interpretation should remain cautious regarding causal inference. Changes in concern patterns may reflect numerous external influences including vaccination rollout, policy changes, and media messaging.

These contextual factors should be more explicitly acknowledged.

Social and economic impacts

This section highlights important competing concerns beyond infection risk.

The findings strongly support the notion that public concerns extended beyond health outcomes.

However, it remains difficult to separate effects arising from pandemic severity versus governmental restriction measures. More explicit discussion distinguishing these factors would strengthen interpretation.

Preventing spread of COVID-19

The section presents important observations regarding perceived rule adherence and public frustration.

The moral framing of non-compliance is particularly interesting and may warrant deeper interpretation.

The authors may also consider whether concern about others' behavior reflects broader concepts such as trust, collective responsibility, or perceived social cohesion.

Government and media

This section captures a notable range of views.

However, government and media concerns often overlap substantially. Participants frequently discuss both simultaneously.

Further explanation regarding how these categories were differentiated analytically would improve clarity.

General concerns

The findings are clearly presented.

However, “general concerns” may partially function as a residual category rather than a distinct conceptual category.

The authors should clarify whether this category represented a meaningful phenomenon or simply uncategorized responses.

Discussion

The discussion appropriately summarizes key findings and links them to prior literature.

However, several interpretations extend beyond direct evidence.

The manuscript occasionally implies explanatory relationships between concerns and infection-prevention behavior despite behavior not being directly examined in this qualitative dataset.

Interpretation should remain aligned with the study design.

The discussion would also benefit from greater attention to: Attrition bias, Limited population diversity, Coding limitations, Potential response bias, Frequency versus psychological importance

These limitations deserve more detailed treatment.

Conclusion

The conclusion generally reflects the results.

However, statements suggesting direct implications for adaptive public health interventions may be somewhat stronger than warranted based on the available evidence.

More cautious wording emphasizing hypothesis generation and informing future work may be preferable.

Reviewer #2: 1. The longitudinal design is valuable, but the potential impact of participant attrition over the five survey waves is not sufficiently addressed. It remains unclear whether those who continued participating differed from those who dropped out, which could influence the observed changes in concerns over time. A clearer assessment of retention bias would strengthen confidence in the reported trends.

2. The qualitative analysis would benefit from greater methodological clarity. Although coding procedures are described, more information on how coding consistency was maintained across time points and how disagreements were resolved is needed. This would help readers better assess the reliability and reproducibility of the findings.

3. Changes in participants’ concerns are interpreted in relation to the evolving pandemic context, yet these links remain largely descriptive. Integrating key events such as lockdowns, vaccination campaigns, or policy changes more directly into the interpretation would provide a stronger explanation for the shifts observed across survey waves.

4. The findings are presented primarily at the overall cohort level, which may mask important differences between population groups. Exploring whether concerns varied according to age, health status, socioeconomic background, or other participant characteristics would enhance the practical and public health relevance of the study.

5. The sample composition limits broader generalizability. The cohort included a relatively high proportion of older adults and individuals with long-term health conditions, while some demographic groups were underrepresented. A more explicit discussion of how these characteristics may have influenced the dominant concerns identified would improve interpretation of the results.

6. While the manuscript references COM-B and PRIME Theory, the connection between the findings and these frameworks could be developed further. Clarifying how the identified concerns relate to specific theoretical constructs would strengthen the study’s contribution beyond descriptive reporting.

7. The analysis focuses mainly on the prevalence of concern categories, providing a useful overview but offering limited insight into how concerns interacted in participants’ lives. Greater attention to the balance between health, social, financial, and emotional concerns would provide a more nuanced understanding of the experiences and behavioral motivations underlying the reported trends.

**********

what does this mean?). If published, this will include your full peer review and any attached files.

If you choose “no”, your identity will remain anonymous but your review may still be made public.

**Do you want your identity to be public for this peer review?**  For information about this choice, including consent withdrawal, please see our Privacy Policy

Reviewer #1: No

Reviewer #2: No

**********

[NOTE: If reviewer comments were submitted as an attachment file, they will be attached to this email and accessible via the submission site. Please log into your account, locate the manuscript record, and check for the action link "View Attachments". If this link does not appear, there are no attachment files.]

To ensure your figures meet our technical requirements, please review our figure guidelines: https://journals.plos.org/plosone/s/figures

You may also use PLOS’s free figure tool, NAAS, to help you prepare publication quality figures: https://journals.plos.org/plosone/s/figures#loc-tools-for-figure-preparation.

NAAS will assess whether your figures meet our technical requirements by comparing each figure against our figure specifications.

Attachment Submitted filename: reviewer comments.docx

Attachment Submitted filename: Review-COVID19.pdf

# Author comment: Author response to Decision Letter 1

**DOI:** 10.1371/journal.pone.0348308.r002

31 Aug 2026

Reviewer 1

1. Title. Long and may benefit from simplification. The phrase “over a two-year period” is informative but somewhat repetitive given the longitudinal context already implied. Consider a more concise title while preserving the key methodological aspect and longitudinal emphasis.

We have amended the title as suggested, removing the phrase ‘over a two-year period’.

2. Abstract. The abstract is well structured and provides a concise overview of objectives, methods, results, and conclusions. However, a numerical inconsistency requires correction.

a. The abstract reports 41,564 coded responses, whereas the Results section later reports 41,568 comments. This discrepancy should be resolved.

Many thanks for highlighting this discrepancy. We have checked our data and corrected the number of responses reported in the results to 41,564.

b. The conclusion section may slightly overstate implications by implying direct effects on future behavioral interventions. Since the analysis focuses on concerns rather than measured behavior, wording should remain more cautious.

We have revised the conclusions in the abstract to ensure that these are more cautious and align more closely with our study design.

3. Keywords. Please use keywords from the Mesh.

We have revised the keywords provided for this manuscript to ensure that these are compatible with MeSH terms.

4. Introduction. The introduction establishes the rationale for studying changing COVID-19 concerns and appropriately distinguishes affective risk perception from broader risk constructs. The literature review is generally comprehensive and logically structured. However, several issues require attention.

a. The opening sentence contains a grammatical issue:

"During pandemics perception of risk can strongly influenced people’s engagement..." This should be revised for clarity and language accuracy.

Thank you for highlighting this error. We have corrected this in the text.

b. The introduction strongly argues that evolving concerns influence infection-prevention behavior, yet the manuscript does not directly measure behavior in the current analysis. The distinction between concern and behavior should be clarified earlier to avoid creating expectations that cannot be addressed by the study design.

We agree that the current framing of the introduction does not make it clear enough that the focus of this study is on understanding changes in concerns, rather than directly assessing behaviour. We have amended the text in several places in the introduction to clarify the focus of this study.

c. The justification for the study would also benefit from a clearer explanation of why existing quantitative longitudinal studies were insufficient and why qualitative content analysis specifically offers additional value.

We have strengthened the rationale for qualitative content analysis in the introduction. We highlight that existing longitudinal surveys typically measured risk perception using structured scales assessing perceived susceptibility, severity or fear. While valuable, these measures cannot reveal the specific concerns underpinning those responses. The present analysis provides insight into the content of concerns and how their relative prominence changed over time, thereby complementing existing quantitative evidence.

5. Method. The COVID-19 Public Experiences (COPE) Study. The longitudinal design is a notable strength and allows examination of changes over time. However, substantial participant attrition occurred across survey waves, declining from 11,113 participants at baseline to 3,827 participants at 24 months. The manuscript does not sufficiently discuss the implications of this reduction. Attrition bias may have substantially influenced the nature of concerns expressed at later time points. Participants remaining in a two-year longitudinal study may systematically differ in motivation, health status, or anxiety profile from those who withdrew. The authors should discuss this limitation more critically and consider whether baseline comparisons between retained and non-retained participants were performed.

We agree that attrition is an important limitation of longitudinal cohort studies. We have provided a breakdown of demographic characteristics of participants at each survey time point in the supplementary materials (Table S1). We have now added additional text in the Strengths and Limitations section acknowledging the retention bias and potential impact of this in more detail.

6. Theoretical approach. The manuscript introduces COM-B and PRIME theory as conceptual frameworks guiding interpretation. Although theoretically relevant, the integration appears relatively superficial throughout the manuscript. The theories are discussed in considerable detail initially, but subsequent analysis remains largely descriptive. The manuscript would be strengthened by demonstrating how individual concern categories align with theoretical constructs. For example: Which concerns correspond to automatic versus reflective motivation? How do competing needs proposed within PRIME theory emerge within the data? Which findings support specific theoretical mechanisms?

We thank the reviewer for this insight and agree that there is currently a disconnect between the theory presented in the introduction and interpretation/discussion of findings. We have included a new subsection in the discussion to clarify how the concern categories identified align with the COM-B and PRIME frameworks.

7. Study population and recruitment. The authors describe recruitment procedures adequately and provide demographic information. However, representativeness remains a major concern. The study population consisted predominantly of: Female participants (69.2%), White British participants (95.8%) and Older adults (>51 years: 68.3%). COVID-19 concerns differed substantially across demographic groups throughout the pandemic. Therefore, the lack of diversity limits broader transferability. The discussion should more critically acknowledge the possibility that the dominant concern patterns identified may largely reflect this specific demographic profile.

We thank the reviewer for this observation. We agree that the demographic profile of the cohort may have influenced the concerns identified. As an analysis of qualitative data, the purpose of this study is to provide in-depth insights into the nature and prominence of concerns arising from the lived experience of those taking part and it is not intended to provide population-level generalisable prevalence data. We have clarified this in the analysis section of the methods and have expanded the discussion and strengths and limitations sections to acknowledge more explicitly that the prominence of concerns relating to personal vulnerability, severe illness and long-term health consequences may reflect the over-representation of older adults, women and people with pre-existing health conditions within the cohort. We also note that concerns particularly relevant to younger populations and ethnic minority communities may be under-represented, and therefore caution against assuming that the findings are representative of the wider UK population.

8. Materials and measures. The open-ended survey question enabled broad capture of participant experiences and concerns. However, the wording: "If you are worried about coronavirus (COVID-19), please tell us what your main concerns are" may unintentionally introduce selection bias. Participants not identifying themselves as “worried” may provide shorter responses or omit concerns entirely. The framing itself could influence the type and intensity of responses generated. The authors should discuss whether this wording may have shaped findings.

The prompt was framed in this way so as not to assume that survey respondents were worried or had concerns about COVID-19, enabling people to report any concerns that they may have in their own words. However, we recognise that this could result in underreporting, and this has been noted in the discussion. We have expanded on this in the relevant sections of the methods and limitations section of the discussion to provide greater clarity.

9. Analysis. The qualitative content analysis approach is appropriate given the volume of responses. However, several methodological issues require clarification.

a. The coding framework was developed from baseline data and then applied deductively to later surveys. Although practical, this strategy risks anchoring later analyses to concerns dominant during the earliest pandemic stage. Novel issues emerging later may have been under-recognized. Further discussion of safeguards against framework rigidity would strengthen confidence in findings.

We recognise the potential risk of framework rigidity. However, the coding framework was not applied in a purely deductive manner. As described in the methods section, ongoing inductive coding was undertaken throughout subsequent survey waves and new categories and subcategories were created when data did not fit the existing framework.

b. Additionally, although a 10% sample underwent verification, no formal inter-coder agreement statistics are reported. Given involvement of eleven coders, further information regarding coding consistency would improve methodological transparency.

We acknowledge that formal agreement statistics were not calculated. Our quality assurance approach was instead based on team-based qualitative coding procedures, including detailed coding guidance, coder training, regular meetings, second-review verification of a random 10% sample, and independent review of the general concerns category. We have expanded the Methods section to describe these procedures more clearly and have acknowledged the absence of formal inter-coder reliability statistics as a limitation.

c. The rationale for reporting detailed coder demographics should also be clarified. If reflexivity was intended, this should be explicitly stated.

We have clarified that coder characteristics were reported as part of a reflexive qualitative approach. As interpretation is influenced by researchers' backgrounds and disciplinary perspectives, we considered transparency regarding the composition of the coding team important as part of our process of ensuring methodological rigour.

10. Results

a. Personal harm from COVID-19. This section clearly describes temporal changes in concern categories. However, frequency of concerns should not necessarily be interpreted as equivalent to psychological importance or intensity. Less frequently reported concerns may still carry substantial emotional significance. Clarification regarding interpretation of frequency data would strengthen the analysis.

Thank you for highlighting this important issue. We have clarified in the results section that frequencies should not be interpreted as a direct measure of psychological importance or emotional intensity relating to specific concerns. Rather, they indicate how commonly specific concerns were mentioned within participants' responses. Less frequently occurring concerns could nevertheless have profound significance for the individuals reporting them.

b. Several participant quotations are useful, although some are relatively long and occasionally interrupt narrative flow. More concise quotations may improve readability.

Thank you for this comment. We have reviewed the results section and reduced the length of several of the longer quotations to improve readability where possible, without losing meaning and context.

c. Harm to others from COVID-19. The findings are well presented and demonstrate an interesting shift from collective concerns toward more individual concerns over time. However, interpretation should remain cautious regarding causal inference. Changes in concern patterns may reflect numerous external influences including vaccination rollout, policy changes, and media messaging. These contextual factors should be more explicitly acknowledged.

We have expanded on this in the discussion section to ensure that a broader range of external influencing factors as well as the characteristics of this cohort are considered in interpreting the findings.

d. Social and economic impacts

This section highlights important competing concerns beyond infection risk.

The findings strongly support the notion that public concerns extended beyond health outcomes. However, it remains difficult to separate effects arising from pandemic severity versus governmental restriction measures. More explicit discussion distinguishing these factors would strengthen interpretation.

We have amended the discussion to acknowledge that the concerns reported by participants could have arisen from both the direct effects of the pandemic and the measures implemented to control transmission, and that these influences are difficult to disentangle. Specifically, we now note that concerns relating to social isolation, education, finances, daily activities, and mental well-being often reflected experiences arising from both the health threat posed by COVID-19 and the associated public health restrictions. We clarify that due to the qualitative nature of the data and the way concerns were reported it is not possible to determine the relative contribution of pandemic severity versus governmental mitigation measures. Rather, the study enables us to understand how participants experienced and balanced these interconnected influences within their lived experience.

e. Preventing spread of COVID-19

The section presents important observations regarding perceived rule adherence and public frustration. The moral framing of non-compliance is particularly interesting and may warrant deeper interpretation. The authors may also consider whether concern about others' behavior reflects broader concepts such as trust, collective responsibility, or perceived social cohesion.

Thank you for this suggestion. We agree that concerns about others not following guidance appeared to extend beyond practical concerns about infection risk and were frequently framed in moral terms, including frustration, unfairness, and loss of confidence in collective efforts to control transmission. We have expanded the discussion to explore this interpretation in greater depth, highlighting how these concerns may reflect broader issues of trust, collective responsibility, social norms, and social cohesion during a public health emergency. We have also strengthened the theoretical link between these concerns about non-compliance to socially oriented motivations and perceptions of personal control within the COM-B and PRIME Theory sub-section of the discussion.

f. Government and media

This section captures a notable range of views. However, government and media concerns often overlap substantially. Participants frequently discuss both simultaneously. Further explanation regarding how these categories were differentiated analytically would improve clarity.

We have revised the manuscript to clarify how these categories were differentiated during analysis in the results section as these concerns were often intertwined in people’s descriptions of their concerns. Comments relating mainly to government decision-making, public health policies, civil liberties, political leadership, or trust in government were coded as government concerns, while comments focusing on the quality, tone, accuracy, volume, or reliability of information and coverage were coded as media concerns. Where participants discussed both topics within a single response, comments could be assigned to multiple categories in line with our coding framework. We have added text to clarify this distinction and acknowledge the overlap between these domains in participants' responses.

g. General concerns

The findings are clearly presented. However, “general concerns” may partially function as a residual category rather than a distinct conceptual category. The authors should clarify whether this category represented a meaningful phenomenon or simply uncategorized responses.

While the General Concerns category included some non-specific responses that could not be allocated to a more specific category, it was not intended solely as a residual coding category. We identified a distinct set of responses characterised by broad uncertainty, concerns about the future trajectory of the pandemic, and apprehension regarding when or whether life would return to normal. These responses reflected uncertainty itself as a salient concern, rather than concerns about a specific aspect of COVID-19. We have revised the manuscript to clarify this point and to emphasise that the category encompassed both non-specific concerns and broader uncertainty-related concerns that represented a meaningful aspect of participants' experiences during the pandemic.

11. Discussion

The discussion appropriately summarizes key findings and links them to prior literature. However, several interpretations extend beyond direct evidence.

a. The manuscript occasionally implies explanatory relationships between concerns and infection-prevention behavior despite behavior not being directly examined in this qualitative dataset. Interpretation should remain aligned with the study design.

Thank you for this observation. We have carefully reviewed the manuscript and revised the wording throughout to ensure interpretations are aligned with the study design. We more clearly distinguish concerns from behavioural outcomes, emphasise that the purpose of the analysis is to understand the nature and evolution of concerns, and avoid implying direct explanatory relationships between reported concerns and infection-prevention behaviours. References to COM-B and PRIME Theory have been retained as theoretical frameworks to aid interpretation of the findings rather than as evidence of behavioural mechanisms demonstrated by the data.

b. The discussion would also benefit from greater attention to: Attrition bias, Limited population diversity, Coding limitations, Potential response bias, Frequency versus psychological importance. These limitations deserve more detailed treatment.

We have significantly expanded on the limitations section of the discussion to provide further detail on these issues, as well as ensuring that our interpretation of findings and conclusions are more aligned with the study aims and design.

12. Conclusion

The conclusion generally reflects the results. However, statements suggesting direct implications for adaptive public health interventions may be somewhat stronger than warranted based on the available evidence. More cautious wording emphasizing hypothesis generation and informing future work may be preferable.

We have re-worded the conclusions to be more cautious, in line with this feedback, noting that findings contribute to understanding how public concerns evolve during a prolonged public health emergency and may help identify issues that warrant attention in future pandemic planning.

Reviewer #2:

1. The longitudinal design is valuable, but the potential impact of participant attrition over the five survey waves is not sufficiently addressed. It remains unclear whether those who continued participating differed from those who dropped out, which could influence the observed changes in concerns over time. A clearer assessment of retention bias would strengthen confidence in the reported trends.

Details of the demographic characteristics of participants at each survey time point is provided in the supplementary materials (Table S1). We have now significantly expanded on the limitations section of the discussion to provide further detail on these issues, as well carefully reviewing the manuscript to ensure that our interpretation of findings and conclusions reflect the study design and population.

2. The qualitative analysis would benefit from greater methodological clarity. Although coding procedures are described, more information on how coding consistency was maintained across time points and how disagreements were resolved is needed. This would help readers better assess the reliability and reproducibility of the findings.

We have expanded on this in the methods, incorporating also feedback from Reviewer 1, to provide greater clarity and transparency around the coding process.

3. Changes in participants’ concerns are interpreted in relation to the evolving pandemic context, yet these links remain largely descriptive. Integrating key events such as lockdowns, vaccination campaigns, or policy changes more directly into the interpretation would provide a stronger explanation for the shifts observed across survey waves.

Thank you for this helpful suggestion. We have strengthened the integration of pandemic context throughout the manuscript. The description of each survey wave in the Methods section to include the dominant SARS-CoV-2 variant, the status of lockdown restrictions, and the timing of the vaccination rollout. We have now incorporated these contextual factors more explicitly into the Results and Discussion when interpreting changes in concerns over time. For example, we now discuss increased concerns relating to clinical vulnerability in the context of reductions in population-level protections; interpret changing perceptions of personal harm alongside vaccination rollout, population immunity, and changes in dominant variants; and consider the influence of shifts in government policy and messaging, including the transition towards a 'Living with COVID-19' approach. We have also expanded the theoretical interpretation of the findings to acknowledge that changes in concern categories occurred within a rapidly changing context characterised by evolving policy measures, vaccination programmes, variants of concern, and communication environments. This provides a more explicit interpretation of how changing pandemic conditions may have shaped the concerns reported by participants while remaining cautious about causal attribution.

4. The findings are presented primarily at the overall cohort level, which may mask important differences between population groups. Exploring whether concerns varied according to age, health status, socioeconomic background, or other participant characteristics would enhance the practical and public health relevance of the study.

Thank you for this helpful suggestion. We agree that understanding variation in concerns across population groups is important for informing targeted public health responses. As the primary aim of this qualitative content analysis was to characterise the nature and evolution of concerns reported by the cohort as a whole, the study was not originally designed to undertake formal subgroup comparisons. However, in response to this comment we conducted additional exploratory descriptive analyses examining the frequency of concerns within each main category according to age group, gender and presence of pre-existing medical conditions at baseline. These analyses identified several noteworthy patterns, including a greater prominence of personal harm concerns among older adults and participants with pre-existing conditions, and relatively greater prominence of concerns relating to harm to others, wider social and economic impacts, and prevention of transmission among younger participants and women. We have added a brief summary of these findings to the Results section and provided the corresponding tables as supplementary material (Tables S4-S6). We have also added a note to the discussion emphasising that these observations are descriptive and hypothesis-generating, and that future research using quantitative longitudinal approaches would be valuable for examining demographic predictors of specific concern categories in greater detail.

5. The sample composition limits broader generalizability. The cohort included a relatively high proportion of older adults and individuals with long-term health conditions, while some demographic groups were underrepresented. A more explicit discussion of how these characteristics may have influenced the dominant concerns identified would improve interpretation of the results.

We have provided further detail in our limitations section to highlight the unique features of our cohort and selective attrition, as well as incorporating a more detailed discussion of how the features of our cohort may have influenced the range of concerns reported, highlighting that the concerns of younger people and those from ethnic minority communities in particular are likely to be under-represented. We also explicitly highlight the need to design future pandemic research in a way that captures the views and experiences of under-served groups in our limitations section.

6. While the manuscript references COM-B and PRIME Theory, the connection between the findings and these frameworks could be developed further. Clarifying how the identified concerns relate to specific theoretical constructs would strengthen the study’s contribution beyond descriptive reporting.

Thank you for this insight. We agree that there is a disconnect between the theory presented in the introduction and interpretation/discussion of findings. We have included a new subsection in the discussion to clarify how the concern categories identified align with the COM-B and PRIME frameworks.

7. The analysis focuses mainly on the prevalence of concern categories, providing a useful overview but offering limited insight into how concerns interacted in participants’ lives. Greater attention to the balance between health, social, financial, and emotional concerns would provide a more nuanced understanding of the experiences and behavioral motivations underlying the reported trends.

Thank you for this valuable suggestion. We agree that understanding how concerns differed between demographic and health-related groups would be highly relevant for public health planning. However, the primary aim of this study was to characterise the nature and evolution of concerns expressed across the cohort as a whole using qualitative content analysis. Participants could report multiple concerns within a single response and concern categories were not designed as mutually exclusive outcome variables for inferential subgroup comparisons. Consequently, detailed analyses of demographic differences were considered beyond the scope of the present paper. We have, however, expanded the Discussion and Limitations sections to acknowledge that the prominence and nature of concerns may have varied across population groups and that future research should explore demographic predictors of specific concerns using quantitative and mixed-methods approaches.

Additional Editor Comments:

The manuscript primarily presents descriptive frequencies of concern categories across survey waves. To strengthen the quantitative component, the authors should consider formally evaluating temporal changes using longitudinal statistical methods, such as mixed-effects logistic regression or generalized estimating equations (GEE), which appropriately account for repeated measurements within participants. In addition, the authors may consider examining whether demographic and clinical characteristics (e.g., age, sex, education, and pre-existing medical conditions) are associated with the likelihood of reporting specific concern categories. Such analyses would provide valuable insights into population subgroups at greater risk of particular concerns and enhance the practical implications of the findings.

Consider adding a Sankey (alluvial) diagram illustrating transitions in participants' primary concern categories across survey waves. Such a figure would provide an intuitive visualization of how concerns evolved over time.

We thank the Editor for these suggestions. We would like to clarify that this study was designed and conducted as a qualitative content analysis of open-ended survey responses, with the aim of characterising the nature, prominence and evolution of concerns expressed by participants in their own words over the first two years of the pandemic, rather than to test hypotheses about predictors of concern categories. Content analysis of this kind is intended to represent the meaning and relative prominence of qualitatively-derived categories within a dataset. Within this context, the resulting frequencies are best understood as a descriptive summary of coded content, not as outcome variables for further quantitative analyses. Applying inferential longitudinal methods such as mixed-effects logistic regression or GEE would fundamentally change the nature of the paper, from a naturalistic meaning-focused qualitative design to a variable-centred quantitative one. We believe this falls outside the scope and aims of the current analysis.

There is also a practical methodological barrier. As participants could report multiple concerns within a single response and be coded into multiple categories simultaneously, concern frequencies do not represent mutually exclusive outcomes suitable for straightforward interpretation using conventional regression approaches. We have clarified this distinction in the methods and discussion sections.

We agree that formal quantitative modelling of concern predictors and transitions would be a valuable direction for future research. We have therefore expanded the Discussion to note that this would require development of structured, quantitative measures of concern, informed by the categories identified inductively here, which could then be analysed using mixed-effects or GEE approaches. We see this as a natural next step building on the present qualitative analysis, rather than an amendment that could be made to the current dataset. Likewise, this would make generating a Sankey diagram challenging, requiring substantial simplification of the qualitative data. We have, however, included in Figure 1 a summary of the percentage of participants reporting one or more concerns in each category at each survey time point, providing a visual overview of change over time that remains consistent with the descriptive nature of the data.

# Editor report: Decision Letter 1

**DOI:** 10.1371/journal.pone.0348308.r003

**Authors:** Jafar Kolahi

21 Sep 2026

Dear Dr. Phillips,

Thank you for submitting your manuscript to PLOS One. After careful consideration, we feel that it has merit but does not fully meet PLOS One’s publication criteria as it currently stands. Therefore, we invite you to submit a revised version of the manuscript that addresses the points raised during the review process.

Please submit your revised manuscript by Nov 05 2026 11:59PM. If you will need more time than this to complete your revisions, please reply to this message or contact the journal office at plosone@plos.org. When you're ready to submit your revision, log on to https://www.editorialmanager.com/pone/ and select the 'Submissions Needing Revision' folder to locate your manuscript file.

- A letter that responds to each point raised by the academic editor and reviewer(s). You should upload this letter as a separate file labeled 'Response to Reviewers'.
- A marked-up copy of your manuscript that highlights changes made to the original version. You should upload this as a separate file labeled 'Revised Manuscript with Track Changes'.
- An unmarked version of your revised paper without tracked changes. You should upload this as a separate file labeled 'Manuscript'.

If you would like to make changes to your financial disclosure, please include your updated statement in your cover letter. Guidelines for resubmitting your figure files are available below the reviewer comments at the end of this letter.

If applicable, we recommend that you deposit your laboratory protocols in protocols.io to enhance the reproducibility of your results. Protocols.io assigns your protocol its own identifier (DOI) so that it can be cited independently in the future. For instructions see: https://journals.plos.org/plosone/s/submission-guidelines#loc-laboratory-protocols. Additionally, PLOS One offers an option for publishing peer-reviewed Lab Protocol articles, which describe protocols hosted on protocols.io. Read more information on sharing protocols at https://plos.org/protocols?utm_medium=editorial-email&utm_source=authorletters&utm_campaign=protocols.

As the corresponding author, your ORCID iD is verified in the submission system and will appear in the published article. PLOS supports the use of ORCID, and we encourage all coauthors to register for an ORCID iD and use it as well. Please encourage your coauthors to verify their ORCID iD within the submission system before final acceptance, as unverified ORCID iDs will not appear in the published article. *Only*  the individual author can complete the verification step; PLOS staff *cannot*  verify ORCID iDs on behalf of authors.

We look forward to receiving your revised manuscript.

Kind regards,

Jafar Kolahi

Academic Editor

PLOS One

Journal Requirements:

If the reviewer comments include a recommendation to cite specific previously published works, please review and evaluate these publications to determine whether they are relevant and should be cited. There is no requirement to cite these works unless the editor has indicated otherwise.

Please review your reference list to ensure that it is complete and correct. If you have cited papers that have been retracted, please include the rationale for doing so in the manuscript text, or remove these references and replace them with relevant current references. Any changes to the reference list should be mentioned in the rebuttal letter that accompanies your revised manuscript. If you need to cite a retracted article, indicate the article’s retracted status in the References list and also include a citation and full reference for the retraction notice.

Additional Editor Comments:

1. Please clarify that the observed changes in concern frequencies represent differences between survey waves and do not necessarily demonstrate changes within individual participants, particularly given the substantial attrition.

2. Please ensure that the abstract and Results consistently distinguish between the number of coded responses, the number of individual participants, and the frequency of concern categories, as participants could report multiple concerns.

3. Please revise the statement in the Introduction suggesting that the study examines changes in concerns at a population level. The non-representative sample and selective attrition do not support population-level generalization.

4. Please clarify that the absence of mutually exclusive concern categories does not, by itself, preclude longitudinal statistical analysis. The decision to retain a descriptive qualitative approach should instead be justified by the study objectives and analytical framework.

5. Please ensure that the exploratory demographic comparisons are consistently described as descriptive findings rather than evidence of demographic predictors of concern categories.

6. Please correct the inconsistency in the corresponding author's email address between the manuscript title page and the Data Availability Statement.

[Note: HTML markup is below. Please do not edit.]

To ensure your figures meet our technical requirements, please review our figure guidelines: https://journals.plos.org/plosone/s/figures

You may also use PLOS’s free figure tool, NAAS, to help you prepare publication quality figures: https://journals.plos.org/plosone/s/figures#loc-tools-for-figure-preparation.

NAAS will assess whether your figures meet our technical requirements by comparing each figure against our figure specifications.

# Author comment: Author response to Decision Letter 2

**DOI:** 10.1371/journal.pone.0348308.r004

14 Oct 2026

Dear Professor Kolahi,

Thank you for the opportunity to revise our manuscript ‘Evolving concerns: A longitudinal content analysis of free-text reports from the UK COVID-19 Public Experiences (COPE) study cohort’ (PONE-D-26-18243R1).

We have responded to the comments from the editor as follows:

1. Please clarify that the observed changes in concern frequencies represent differences between survey waves and do not necessarily demonstrate changes within individual participants, particularly given the substantial attrition.

We have amended the text in the analysis section on page 11 (lines 258-259) to explicitly clarify this: ‘These analyses were intended to provide contextual insight rather than formal subgroup comparisons, highlighting differences in concerns reported at each survey wave overall as opposed to changes at an individual level.’

2. Please ensure that the abstract and results consistently distinguish between the number of coded responses, the number of individual participants, and the frequency of concern categories, as participants could report multiple concerns.

The abstract has been amended to ensure that it is clear that respondents could report multiple concerns, and to clearly indicate where findings relate to frequency of concerns within a category or number of respondents reporting concerns within each category. We have edited the abstract to ensure that it remains within the 300-word limit. We have also reviewed and updated the results section to ensure that text and tables clearly indicate where frequency of concerns vs. number of respondents is indicated. These amendments have been marked using track changes in the abstract and results sections of the manuscript.

3. Please revise the statement in the Introduction suggesting that the study examines changes in concerns at a population level. The non-representative sample and selective attrition do not support population-level generalization.

Thank you for this observation. We have revised the Introduction to remove references to ‘population-level’ shifts in concerns and instead describe the study as examining changes in concerns reported within a large longitudinal community cohort.

4. Please clarify that the absence of mutually exclusive concern categories does not, by itself, preclude longitudinal statistical analysis. The decision to retain a descriptive qualitative approach should instead be justified by the study objectives and analytical framework.

We have revised the manuscript to clarify that our decision to adopt a descriptive qualitative approach was driven by the study aims and content analysis approach, which sought to characterise the nature and evolution of concerns expressed by participants, rather than to model predictors or transitions between concern categories. We have amended the Analysis and Strengths and Limitations sections to reflect this.

5. Please ensure that the exploratory demographic comparisons are consistently described as descriptive findings rather than evidence of demographic predictors of concern categories.

Thank you for this comment. We have reviewed the manuscript to ensure that we consistently describe these findings as exploratory descriptive patterns observed within the cohort, and have avoided language implying prediction, association, or causal inference. Additional clarification has been added to the Analysis, Discussion, and Strengths and Limitations sections to emphasise that these findings are hypothesis-generating only.

6. Please correct the inconsistency in the corresponding author's email address between the manuscript title page and the Data Availability Statement.

Many thanks for highlighting this, we have corrected the error in the Data Availability Statement.

Yours faithfully,

Dr Rhiannon Phillips

Attachment Submitted filename: Response to Reviewers.docx

# Editor report: Decision Letter 2

**DOI:** 10.1371/journal.pone.0348308.r005

**Authors:** Jafar Kolahi

22 Oct 2026

Evolving concerns: A longitudinal content analysis of free-text reports from the UK COVID-19 Public Experiences (COPE) study cohort

PONE-D-26-18243R2

Dear Dr. Phillips,

We’re pleased to inform you that your manuscript has been judged scientifically suitable for publication and will be formally accepted for publication once it meets all outstanding technical requirements.

Within one week, you’ll receive an e-mail detailing the required amendments. When these have been addressed, you’ll receive a formal acceptance letter and your manuscript will be scheduled for publication.

An invoice will be generated when your article is formally accepted. Please note, if your institution has a publishing partnership with PLOS and your article meets the relevant criteria, all or part of your publication costs will be covered. Please make sure your user information is up-to-date by logging into Editorial Manager at Editorial Manager® and clicking the ‘Update My Information' link at the top of the page. For questions related to billing, please contact billing support.

If your institution or institutions have a press office, please notify them about your upcoming paper to help maximize its impact. If they’ll be preparing press materials, please inform our press team as soon as possible -- no later than 48 hours after receiving the formal acceptance. Your manuscript will remain under strict press embargo until 2 pm Eastern Time on the date of publication. For more information, please contact onepress@plos.org.

Kind regards,

Jafar Kolahi

Academic Editor

PLOS One

# Editor report: Acceptance letter

**DOI:** 10.1371/journal.pone.0348308.r006

**Authors:** Jafar Kolahi

PONE-D-26-18243R2

PLOS One

Dear Dr. Phillips,

I'm pleased to inform you that your manuscript has been deemed suitable for publication in PLOS One. Congratulations! Your manuscript is now being handed over to our production team.

At this stage, our production department will prepare your paper for publication. This includes ensuring the following:

* All references, tables, and figures are properly cited

* All relevant supporting information is included in the manuscript submission,

* There are no issues that prevent the paper from being properly typeset

You will receive further instructions from the production team, including instructions on how to review your proof when it is ready. Please keep in mind that we are working through a large volume of accepted articles, so please give us a few days to review your paper and let you know the next and final steps.

Lastly, if your institution or institutions have a press office, please let them know about your upcoming paper now to help maximize its impact. If they'll be preparing press materials, please inform our press team within the next 48 hours. Your manuscript will remain under strict press embargo until 2 pm Eastern Time on the date of publication. For more information, please contact onepress@plos.org.

You will receive an invoice from PLOS for your publication fee after your manuscript has reached the completed accept phase. If you receive an email requesting payment before acceptance or for any other service, this may be a phishing scheme. Learn how to identify phishing emails and protect your accounts at https://explore.plos.org/phishing.

If we can help with anything else, please email us at customercare@plos.org.

Thank you for submitting your work to PLOS One and supporting open access.

Kind regards,

PLOS One Editorial Office Staff

on behalf of

Dr. Jafar Kolahi

Academic Editor

PLOS One
